# A Student's Guide to Randomization Statistics for Multichannel Event-Related Potentials Using Ragu

**DOI:** 10.3389/fnins.2018.00355

**Published:** 2018-06-19

**Authors:** Marie Habermann, Dorothea Weusmann, Maria Stein, Thomas Koenig

**Affiliations:** ^1^Translational Research Center, Department of Psychiatric Neurophysiology, University Hospital of Psychiatry Bern, University of Bern, Bern, Switzerland; ^2^Department of Clinical Psychology and Psychotherapy, University of Bern, Bern, Switzerland

**Keywords:** Ragu, randomization statistics, ERP, N400, EEG, microstates

## Abstract

In this paper, we present a multivariate approach to analyze multi-channel event-related potential (ERP) data using randomization statistics^1^. The MATLAB-based open source toolbox *Randomization Graphical User interface* (Ragu) provides, among other methods, a test for topographic consistency, a topographic analysis of variance, t-mapping and microstate analyses. Up to two within-subject factors and one between-subject factor, each with an open number of levels, can be defined and analyzed in Ragu. Ragu analyses include all sensor signals and no a-priori models have to be applied during the analyses. Additionally, periods of significant effects can be controlled for multiple testing using global overall statistics over time. Here, we introduce the different alternatives to apply Ragu, based on a step by step analysis of an example study. This example study examined the neural activity in response to semantic unexpected sentence endings in exchange students at the beginning of their stay and after staying in a foreign-language country for 5 months.

## 1. Introduction

Imagine a team of neuroscientists who want to know how a particular cognitive act is represented in terms of brain activity. They developed a new experimental design, which involved the use of evoked multichannel scalp electromagnetic field potentials, to test this hypothesis. The data has now been collected and processed according to the established standards. They now have single subject averaged event-related potentials. The next step in their analysis is to test their hypotheses statistically. These hypotheses do not allow them to make concise predictions about how their experimental manipulations will affect their recorded data. How should they proceed? Limiting their analyses to the few most plausible hypotheses increases the risk of missing or misrepresenting the “true” effect. On the other hand, examining every point in time and space will result in a very large number of tests, of which an unknown fraction may be significant due to multiple testing. In addition, as the measurements are not independent, results which are significant will be, to a large extent, redundant. Alternatively, the researchers may choose to apply a particular model that reduces the size of the data, but they would have to assume a-priori that the model and model parameters chosen to reduce the data were appropriate. This may be difficult, but nevertheless, results and conclusions will vary depending on the a-priori assumptions.^1^

**Figure 1:**
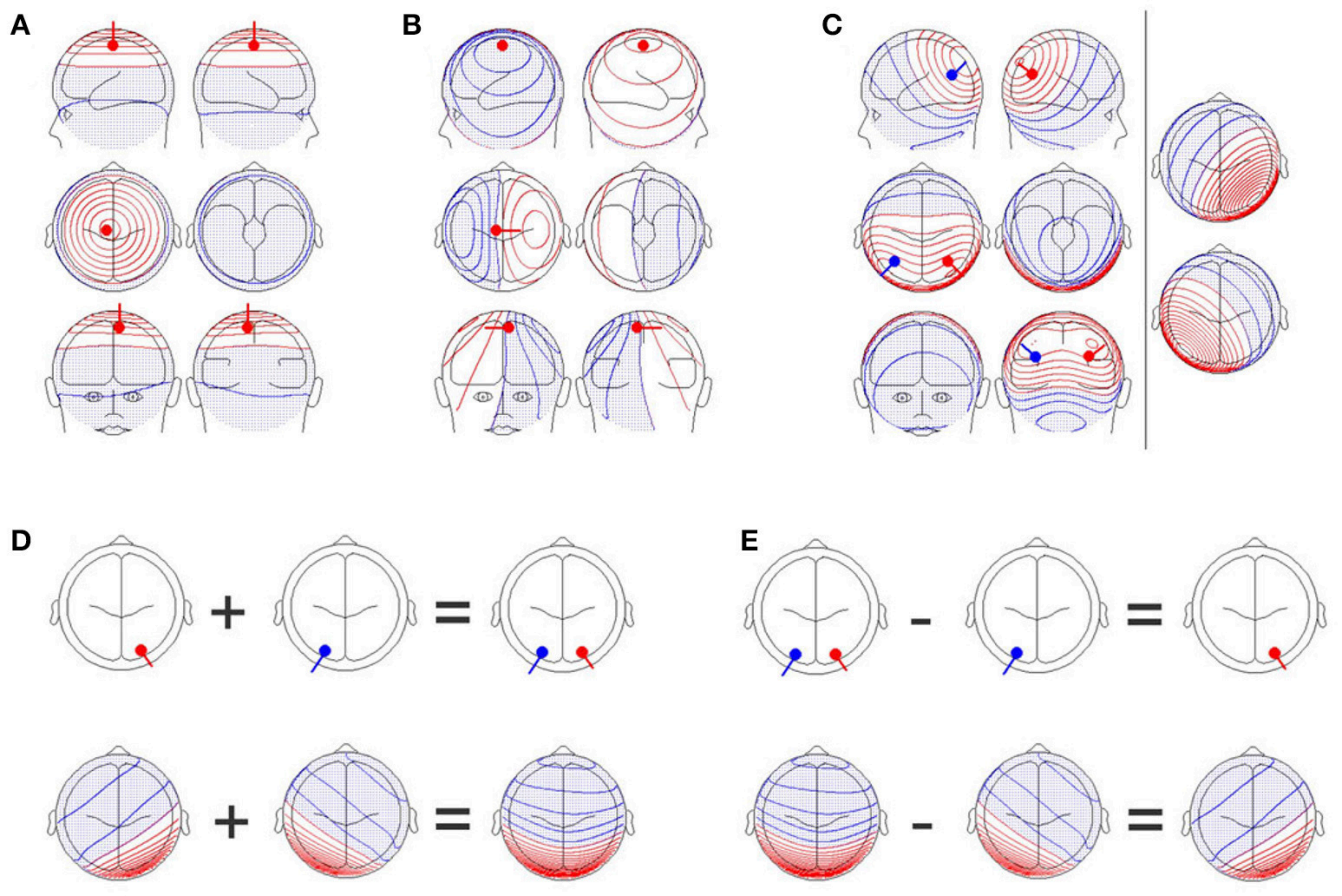
**(A)** A single dipole close to the electrode position, Cz, with an orientation perpendicular to the scalp. Upper row: Images of the left (upper left graph) and right (upper right graph) sides of the head. Middle row: Images from the top (middle left graph) and bottom (middle right graph) of the head. Lower row: Images of the front (lower left graph) and back (lower right graph) of the head. Positive values are shown in red while negative values are shown in blue and with dots. **(B)** A single dipole at the same position as in **(A)**, but the orientation of the dipole has been changed by 90 degrees. The scalp field has drastically changed. **(C)** Two simultaneously active, symmetrical dipoles in the left and right parietal cortex are simulated. The right section of the figure separately shows the scalp field generated by the red and blue dipoles seen in the left section. The left section also shows the scalp field obtained by combining both dipoles. The resulting scalp field shown on the left is the sum of the two scalp fields shown on the right part of the figure. **(D)** Additivity and **(E)** subtractivity of EEG maps. The upper row shows the location of the sources, and the lower row shows the resulting EEG scalp field maps. All graphs were created with the BESA dipole simulator.

A reasonable solution to this problem is to adopt a multivariate approach. This is in line with the fact that scalp electromagnetic field potentials are by nature multivariate. The electromagnetic signals produced by neuronal events in the brain reach the scalp surface through electromagnetic volume conduction. Electromagnetic volume conduction acts as a spatial low-pass filter, such that even a single, and tightly localized event in the brain will lead to scalp potential differences between almost any two scalp positions (Figures 1A,B). Since the scalp fields of several sources are additive (Figure 1C), contrasting scalp fields yields direct evidence for contrasting source configurations (Figures 1D,E). By considering the scalp electromagnetic field (which contains all the information on the differences between all possible pairs of scalp positions) as the basic, multivariate phenomenon to analyze, we do justice to the physical background of the data that we deal with. At the same time, we can thereby avoid problems associated with multiple testing across channels. Thus, procedures that allow statistical inferences based on comparisons of scalp electromagnetic fields are needed.

The software toolbox “Randomization Graphical User Interface” (Ragu) presented in this paper implements such statistical inferences based on scalp electromagnetic fields. Ragu was primarily developed for the analysis of averaged multichannel event-related potentials (ERPs) involving multiple subjects, and allows for two within-subject (repeated measures) factors and one between-subject factor. The number of levels of each factor is not limited. The following sections will demonstrate typical elements analyzed during an ERP study, and will address the following aims^2^:

- Importing the Data (section 2.2), ensuring that it is correctly represented (section 2.3.1), and screening for outliers (section 2.3.2)
- Defining the experimental design (section 2.4)
- Testing, time-point by time-point, for evidence of communality of the scalp field data across subjects (section 4.2)
- Testing for topographic differences by time-points, or within pre-selected analysis periods, and eventual corrections for multiple testing across time (section 4.3)
- Testing for differences in latencies of specific, spatially defined ERP components (microstate analysis, section 4.4.1)

## 2. Preparing the analysis

To introduce how Ragu can be applied, we will use an example data set, collected within a study by Stein et al. (2006). It set out to investigate neuronal plasticity in the language domain by the ERP effects during active German language learning. German sentences were visually presented, and ERPs were recorded in native English-speaking exchange students living in Switzerland. The focus of the present study is on semantic integration implemented in an N400 type experiment, where the last word of the sentence either fit or violated the semantic expectancy created by the preceding parts of the sentence (Kutas and Hillyard, 1980). The sentences presented had either a congruent ending, like “The wheel is round” or an incongruent ending, like “The garden is shy.” We will call this experimental factor “expectancy” because depending on the congruence, the sentence ending was either expected or unexpected by the participants. To assess the effects of language acquisition, the participants underwent the recording of N400 ERP responses at the beginning of their stay in Switzerland and 5 months after the first recording. We will refer to this second factor as “day.” Thus, we have a 2 × 2 design with four conditions, namely “expected day 1,” “unexpected day 1,” “expected day 2,” and “unexpected day 2.”

Sixteen subjects participated in the study^3^. The subjects were right-handed English-speaking exchange students [mean age: 16.9 years (range 16–18 years); 4 males, 12 females]. None of the participants had made any prior systematic attempts to learn German. They all participated in a German language course during their first three weeks in Switzerland. ERPs were recorded from 74 scalp locations with a 250 Hz sampling rate, from the stimulus onset until 1,000 ms post-stimulus. Artifact rejection and averaging across trials were performed according to standard procedures. The data was recomputed to average reference and band-pass filtered between 1.5 and 30 Hz. For further details on data acquisition, experimental setup and study group, please refer to Stein et al. (2006). The data which was analyzed in this study is available in the Supplementary Material (“Data Sheet 1.zip”).

### 2.1. Obtaining the program

Ragu is available for download free of charge. There are pre-compiled versions of the program for Windows and OSX that do not require a MATLAB installation. For MATLAB users, the source code is also available. For these users, it is also possible to access and manipulate Ragu data files directly, as Ragu stores all results in the MATLAB format. The download links can be found on the corresponding author's website.

### 2.2. Importing the data

First of all, we need to import the pre-processed ERP data for our example study. Ragu allows users to import the data of all subjects and conditions in a single step. However, since this requires naming of data files in a specific way, the proper way of doing this will be specified subsequently.

#### 2.2.1. Structuring the raw data for import

All data files to be imported need to be in the same folder. A single file is required for each subject and condition. The required format of each data file is ASCII plain text, organized as time × channel matrices, where the positions of the time instances and channels are the same for all files. The names of these files have to follow a particular and standardized scheme. Firstly, a common label at a defined position in the file name must identify all data files associated with a given subject across all conditions, and secondly, a common label at a defined position in the file name must identify all data files associated with a given condition across all subjects. Thus, there is:

1. A unique identifier of the subject. In our data, this is: S01 for subject one; S02 for subject two, etc.
2. A unique identifier of the experimental condition. In our data, C1 represents “expected day 1” and C2 represents “expected day 2.” F1 represents “unexpected day 1” while F2 represents “unexpected day 2.” The “C” in “C1” indicates a correct sentence ending, while the “F” in “F1” represents a false sentence ending.

It is crucial that the chosen labels for the different subjects and conditions, as well as their positions stay the same. However, the order and labels can be chosen differently. According to this convention, the sample data files associated with conditions C1, C2, F1, and F2 of subject one were labeled *S01_C1.asc, S01_C2.asc, S01_F1.asc*, and *S01_F2.asc*.

**Figure 2:**
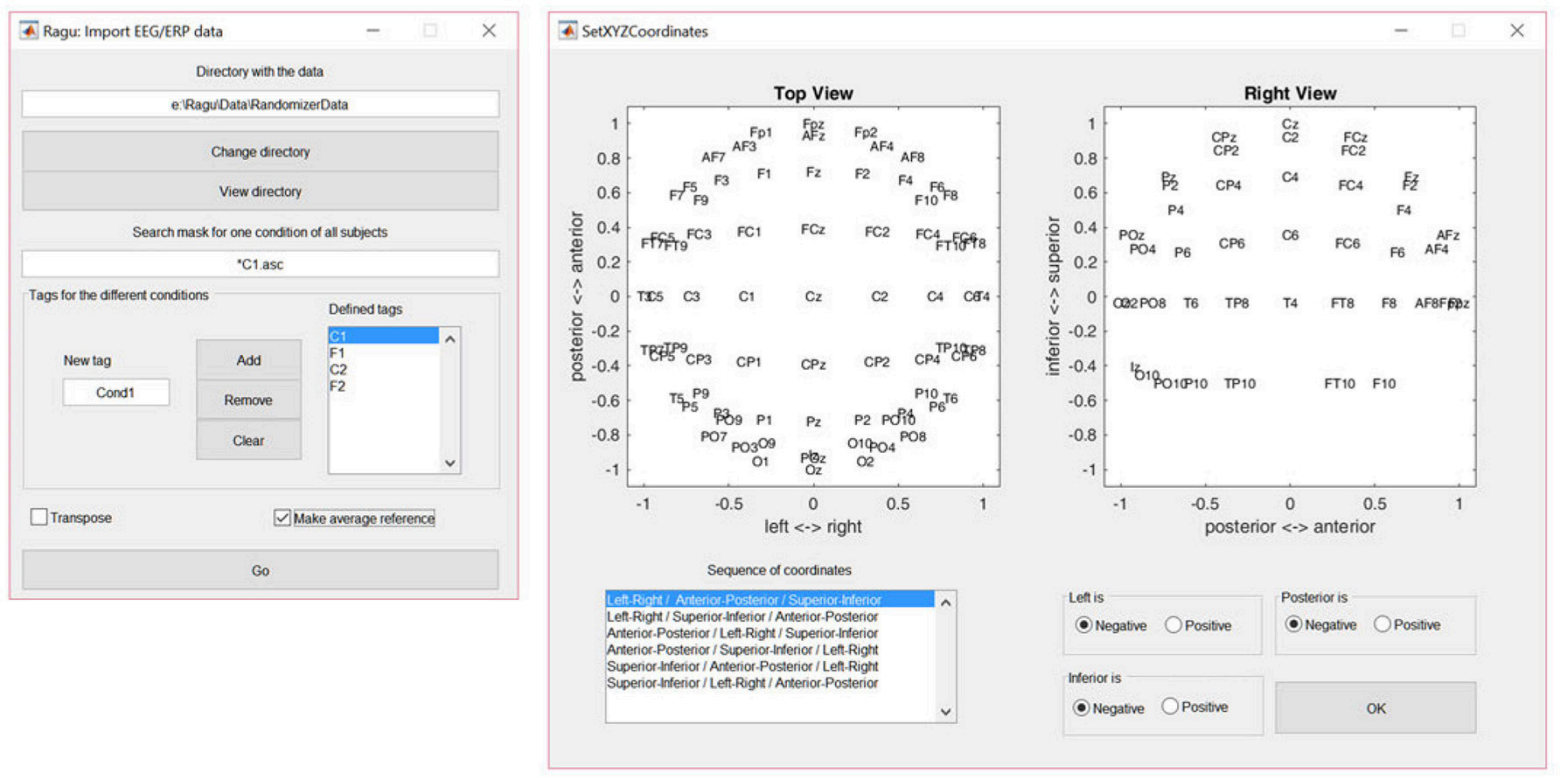
**Left:** The Ragu data import dialog window. The defined tags represent the four conditions considered in the study. The search expression enables the identification of all subjects to be imported. **Right:** The Montage dialog window. The channel coordinates can be visually verified and adjusted if the channel positions are set incorrectly.

The dialog window for importing data is shown in Figure 2. The user is requested to indicate the folder in which the data files are located. To obtain a list of all subjects, a file search expression that identifies all subjects for one of the conditions needs to be provided (see the left panel in Figure 2). Finally, the user needs to specify the labels for all conditions. When the import is completed, Ragu will indicate the number of frames and channels that have been imported. If these numbers are incorrect because the data was organized as channel × time matrices instead of time × channel matrices, there is a check box in the import dialog window to transpose the data while importing (see Figure 2). It is also possible at this point to re-compute the data to average reference, which is strongly recommended for all ERP analyses in Ragu (Michel et al., 2009).

#### 2.2.2. Montage

To later visualize the results as spatial maps, Ragu will ask for the so-called montage, which is the location of the channels on the scalp. The user needs to import an ASCII-file with the position information of the channels (“Coordinates.xyz” in the Supplementary Material). Afterwards, the orientation of the maps can be verified and adjusted manually using the dialog window called “Set XYZ-Coordinates” (see the right side of Figure 2).

### 2.3. Data tools

#### 2.3.1. Data inspection

The advantage of importing data as ASCII-files is that there are few compatibility issues, but this increases the possibility of misrepresenting the data after a seemingly successful import. Therefore, Ragu offers a basic functionality to test if the data has been imported correctly. Before starting the analysis of our example data, we check if the data is correctly represented in Ragu. To do so, select “Data Inspection” in the menu option “Data.” To verify that the potential maps and traces are equal to the ones found during pre-processing, select one or multiple subjects and press “Show.” If more than one subject is selected, the mean of all selected cells will be shown. It is possible to obtain a spatial map view by clicking on the resulting butterfly plots.

#### 2.3.2. Outlier detection

Important basic terms:

- **State space representation:** The channel x time data matrix is represented in an n-dimensional space, where n is the number of electrodes, and each axis of this space represents the voltage at a given electrode. Each moment in time is thus represented by a point in this n-dimensional space. This high-dimensional data representation can be projected onto lower (e.g. two-dimensional) spaces to permit visualization.
- **Multidimensional scaling (MDS):** This is a method for visualizing similarities among datasets in a low-dimensional space. Each dataset is represented by a point, and the distances between these points are approximately inversely proportional to the similarity among the corresponding datasets.
- **Global Field Power (GFP):** This is an index of the overall voltage differences across all channels. It is equivalent to computing the standard deviation across channels.
- **Microstates:** These are continuous periods of time with quasi-stable spatial configurations of the measured scalp fields.

Our aim is to analyze data from people who represent the general population and to exclude extreme values which cannot be generalized to a larger sample. In the outlier detection step, subjects showing substantial disparity from the mean of all the examined subjects can be detected and eliminated. To visually guide the detection of such outliers, *Multi-Dimensional Scaling* (MDS) is used. With this method, high-dimensional spaces can be downscaled into lower dimensional ones and are thus easier to interpret. To detect outliers, the data of each subject (i.e., all channels and time points of all conditions) is arranged in a one-dimensional vector, thus yielding one vector per subject. Next, a matrix of correlations is computed for all those vectors. The MDS algorithm then computes, for each subject, a set of coordinates. These coordinates are chosen in such a way that the Euclidean distances between all pairs of coordinates optimally represent the correlation among the ERP data of the corresponding subjects, with higher correlations resulting in smaller distances. The MDS algorithm then computes, for each subject, a set of coordinates, choosing these coordinates in a way that the Euclidean distances between all pairs of these coordinates optimally represent the correlation among the ERP data of the corresponding subjects, with higher correlations resulting in smaller distances.

**Figure 3:**
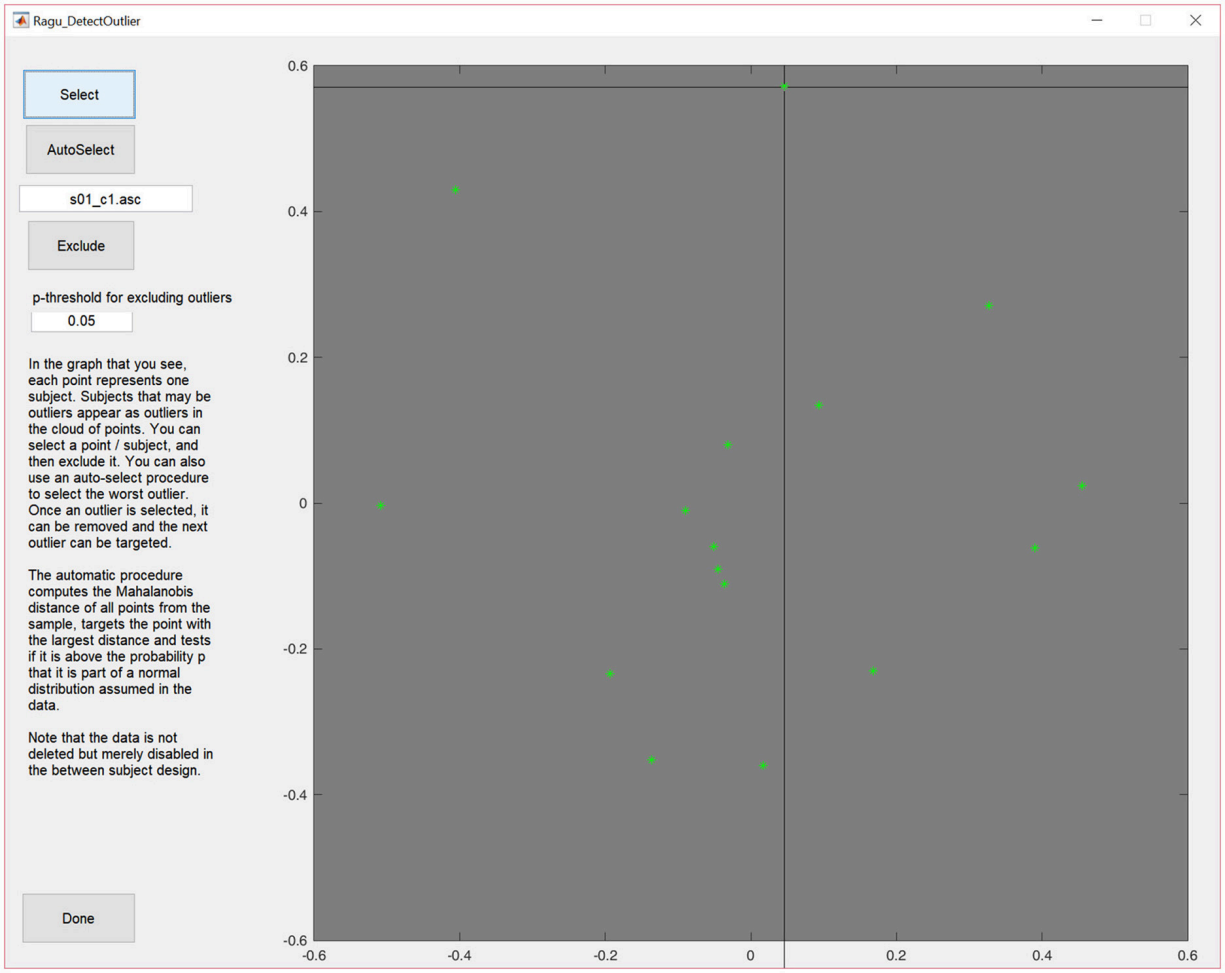
MDS output of the correlation matrix including data from all subjects. The middle of the graph (value = 0) indicates the mean value of the data across all subjects and conditions. The closer a dot is to the center, the more the data of that particular subject resembles the mean of the data of all the other subjects, and the further a dot is away from the center, the less it resembles the mean of the data from the other subjects. Outliers can be selected and excluded from subsequent analysis.

To access the outlier detection tool in Ragu, click on “Data→Outlier detection.” In the resulting graph, and as explained above, each dot represents, as explained above, one subject, and can be selected and excluded (Figure 3).

The display will then be updated without the eliminated subject. Furthermore, there is an (experimental) option to auto-select outliers. It eliminates potential outliers based on an algorithm proposed by Wilks (1963) that uses the Mahalanobis distance among the displayed points to identify cases that are unlikely to be part of the normal distribution.

In our case, no extreme outliers seem present and the auto-select option did not eliminate any subject. We can therefore continue our analysis without excluding any subject. Note that outlier detection in Ragu is essentially an educated means to obtain a visual overview of the data, but the user determines what should be considered as an outlier. This obviously requires a more detailed inspection of the data from each excluded subject and the judgment of someone familiar with the type of data under analysis.

#### 2.3.3. Additional tools

Ragu offers the possibility to filter and\or baseline-correct the ERP data. Thus, the user can apply this baseline correction if pre-stimulus information is eliminated from the data. It is also possible to apply IIR high- and low-cut filters, and a notch filter. The processed data can be reviewered using the “Data-inspection” (see section 2.3.1). Data filtering can also be undone after filters are applied. By selecting the option “Filter Specs,” the information on how the data has been filtered and processed can be reviewed. As our example data is already sufficiently pre-processed and does not contain pre-stimulus measurements, we will not use these tools in our example.

### 2.4. Design

After the data is imported and reviewed, the next step is to define the experimental design. It is possible to define up to two factors for a within-subject design and one factor for a between-subject design. In our example, “expectancy” and “day” are the within-subject factors. If two within-subject factors are set, the levels of these factors have to be orthogonal. If there is only one factor, it can be categorical, rank-order scaled, or interval-scaled.

**Figure 4:**
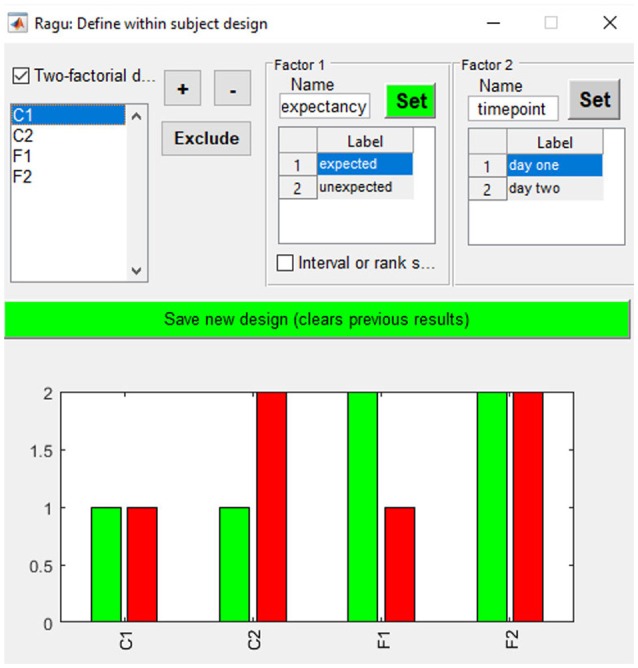
Within-subject design with two orthogonal factors in Ragu. On the upper right side, the two factors and their particular levels (1, 2) are shown. On the bottom, the assignment of conditions to the factor level values is shown. Use “+” and “−” to increase/decrease the assigned factor level.

As our example does not compare distinct groups but factor levels within a group, we define them using the “Design→Within Subject Design” menu. After providing the factor name, the user must click on the “Set” button to define the different factor levels. In our study, factor one is “expectancy” with the factor levels “expected” and “unexpected.” Factor two is “day” with the factor levels “day 1” and “day 2” (see Figure 4). It is also possible to define a between-subject factor in Ragu (menu item “Design→Between Subject Design”). Here, the groups have to be named, and values have to be assigned to the different groups. However, for our example, this is not necessary, and a detailed description will not be provided here. For more details on the between-subject design, see Koenig et al. (2011).

## 3. Basic concepts

Before we go into a step-by-step demonstration of a typical analysis using Ragu, we will introduce a method for graphically representing both the procedures employed and results obtained using Ragu. We will refer to this as a state-space representation. How it works will be described subsequently.

### 3.1. State space representation

**Figure 5:**
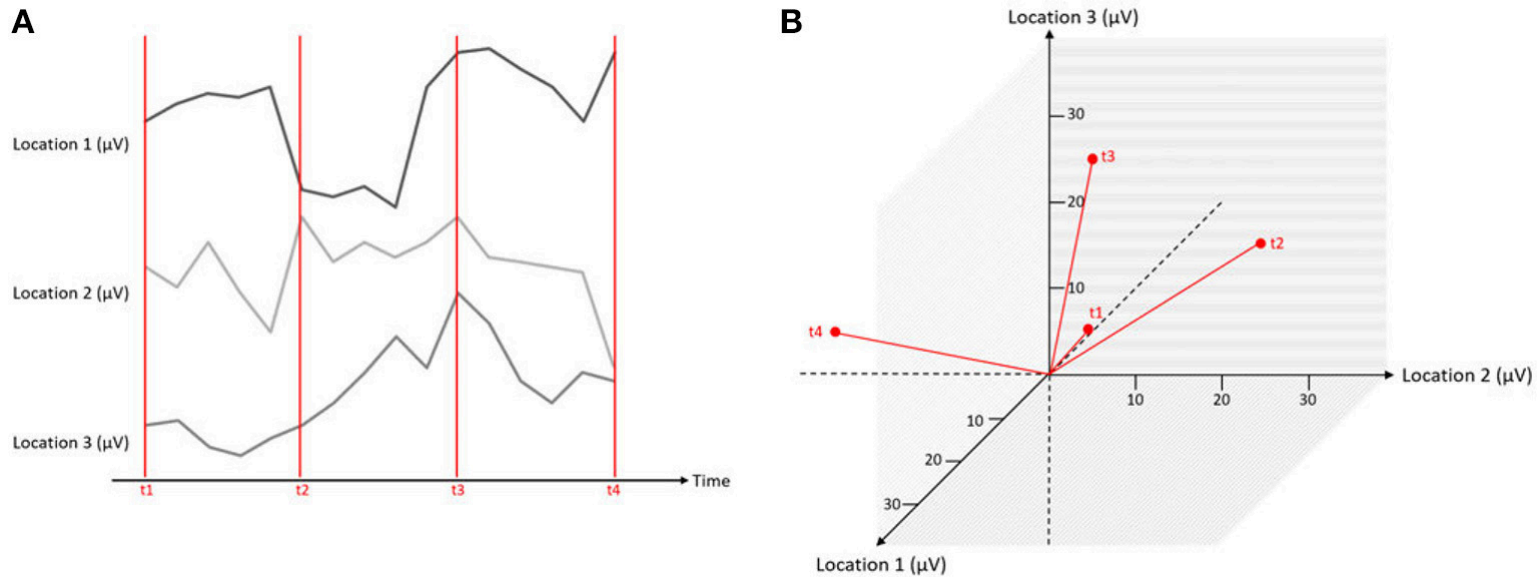
Simultaneous measurements at three locations expressed in one graphical representation. The red lines mark different time points of measurements (t1–t4), and the black lines indicate the microvolt values at three different channels. On the left **(A)**, the data is displayed as separate traces of each channel. On the right **(B)**, the data is displayed in a state space representation.

Let us assume that we have measured the EEG at three scalp locations against a common reference electrode (that is by definition, zero), yielding three data channels. A typical way of displaying such data is to plot the voltage of each channel as a function of time, yielding typical pictures of EEG “traces” (Figure 5A). However, instead of one graph per channel, the information can be combined into a single, three-dimensional representation. In this representation, each channel corresponds to one axis of an orthogonal coordinate system. For a given measurement point, the data from the three channels yield three coordinates on the three orthogonal axes and can be plotted as a point in a three-dimensional space (Figure 5B). Thus, each measurement point of the three-channel dataset yields a point, and the entire dataset is consequently represented by a three-dimensional “object.” This representation has a series of properties that will be relevant for the remainder of this article.

- Each point in this state-space representation represents all measured channels at a given time point, and can therefore be represented as a topographical map.
- The origin of the coordinate system is by definition zero, and therefore, corresponds to the recording reference that is also by definition zero. Re-computing the data against another reference thus corresponds to a parallel translation of all points in space that does not affect its shape.
- Re-referencing the data to average reference is equivalent to centering the points to the origin of the coordinate system.
- The distance between a point and the origin is proportional to the overall strength of the observed scalp field. For data referenced against the average reference, this takes us (after a correction for the number of channels) to the definition of the so called Global Field Power (GFP, Equation 1):
  (1) $$\begin{array}{l} GFP=\sqrt{\frac{\overset{n}{\underset{j=1}{\sum }}{\left(v_{j}-\overset{-}{v}\right)}^{2}}{n}}, \end{array}$$
  where *j* is the channel index, *v*_*j*_ is the voltage measured at channel *j*, $\overset{-}{v}$ is the mean voltage value across all channels (i.e., the average reference) and *n* is the number of channels. The GFP is reference free and can be used to quantify the strength of a map across all channels. Mathematically, computing the GFP is equivalent to computing the standard deviation of the voltage values across all channels (Lehmann and Skrandies, 1980).
- The difference vector between any two points A and B in this state-space representation corresponds to the difference map between the two maps represented by A and B. This vector is thus equivalent to the scalp field produced by all observable sources that differed between the two maps represented by A and B.
- The distance between two points A and B, i.e., the GFP of the difference map A-B (see an example in Figure 1E) is proportional to the overall strength of the scalp field produced by those sources that are different between the maps A and B. If maps A and B correspond to different conditions, the GFP of the difference map will provide a global index of the amount of difference in electromagnetic brain activity (see section 4.3).
- We are free to rotate the obtained three-dimensional object to obtain “views” that we find particularly informative.

EEG recordings usually have more than three channels, which makes the vector-space of the state-space representation more than three-dimensional. However, this does not change the properties of the state-space representations listed above. We can use these properties to deduce important arguments for the analysis and interpretation of our results. In particular, we can use the GFP of difference maps as a global index of difference in brain electrical activity, and we can rotate high-dimensional state-space representations of multichannel data in meaningful ways to obtain visually accessible (usually two-dimensional) and informative representations of their dynamics across time and experimental manipulations.

We will use this form of representation of multiple measurements to introduce different tests performed with the Ragu toolbox, such as the Topographic Consistency Test (section 4.2) and the Topographic Analysis of Variance (section 4.3). The parameters displayed in the state space representation are thus typically rotated versions of the potential values of all channels.

### 3.2. Randomization statistics in general

The following section will explain the statistical approach used in the Ragu toolbox. As already indicated in its name, Ragu uses Randomization statistics, meaning that -similar to other statistical methods- it can be used to test results in terms of the plausibility of a competing null hypothesis. In contrast to other statistical methods, randomization statistics can be applied when a theoretical distribution of some extracted variable is not easily accessible and needs to be computationally estimated. Thus, to test for the probability of the null-hypothesis, the comparison of the observed data is performed against results drawn from randomized data, because the randomized data is hypothesized to represent data in which no effect of a systematic variation of some relevant condition is present. Randomization statistics have a similar statistical power as parametric statistics if the distribution of the data meets the requirements for parametric statistics. If the distribution of the data requires the application of non-parametric statistics, randomization statistics typically have a better statistical power than non-parametric statistics (Manly, 2007).

To illustrate this principle, we use an example of Michel et al. (2009). To test the hypothesis that “stupid farmers have bigger potatoes,” one may pick a smart and a stupid farmer and collect a random bag of potatoes from each of them. Next, the average potato size for the two samples and their difference value is calculated. It may indeed occur that the stupid farmer's potatoes are bigger on the average. However, this result may be due to chance, because the difference in the average potato size may be within the range of differences that one would expect by chance, given the overall variability of potato sizes. To test with randomization statistics whether the difference in potato sizes is significant, the potatoes in both bags are mixed and then randomly reassigned to the bags of the smart and the stupid farmer. Again, the average potato size is calculated for each bag, as well as the difference in average sizes. This procedure (mixing the potatoes in the bags and computing the difference in their average sizes) is then repeated several times, yielding, as a result, a distribution of random average potato size differences. Now the probabilities of obtaining an average potato size difference as the one observed originally and the null-hypothesis being true can be estimated by counting the cases in which the randomly obtained average potato size differences were equal or larger than the observed one.

Thus, to test the informational content of some predictor that is assumed to explain a relevant part of the variance in some measured data, the randomization procedure eliminates any potentially systematic link between the predictor and the data. This yields a distribution of the variance explained by the predictor under the null hypothesis. This distribution then serves to test how likely it is that the variance explained by the predictor in the actually observed data is compatible with the null-hypothesis. Depending on this probability, the null-hypothesis may then eventually be accepted or rejected. For ERP analyses, there are many options for applying randomization statistics. In the subsequent sections, some of them will be presented.

## 4. Analyses

### 4.1. Randomization options

Before commencing with the data analyses, some settings can be adjusted according to the needs of the user. The parameters set in this step will be applied to all the subsequent tests. However, it is possible for the user to return to these choices and readjust the settings if necessary.

#### 4.1.1. Data normalization

**Figure 6:**
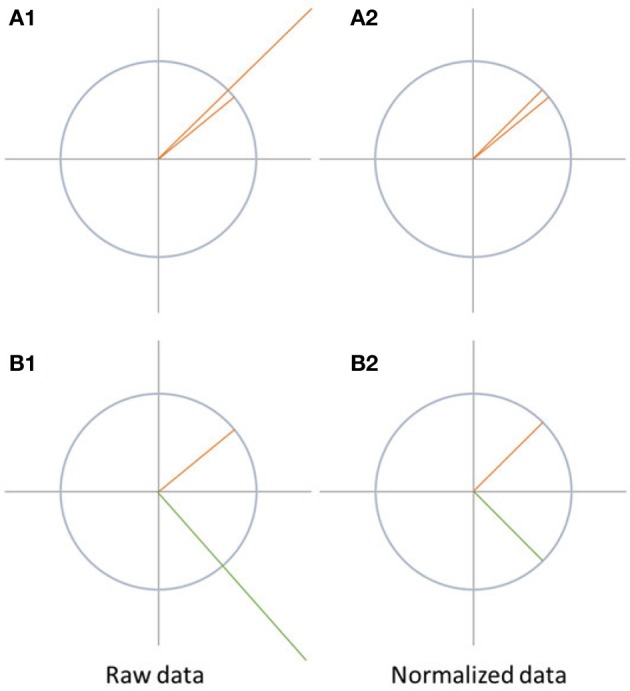
Visualization of data normalization. On the left side, the raw data is shown while the data after normalization is shown on the right side. The differences between A1 and A2 disappear after normalization. In case of B1 and B2, the differences between the two signals are not only due to difference in the strength of the signal, but also because the state-space vectors have a different orientation. Therefore, the difference between the signals remains after normalization.

Map differences between two conditions can be observed due to different reasons, as illustrated in Figure 6. They can be produced by a proportional change in strength in all active sources (case A1 in Figure 6). Alternatively, they may result from a different spatial distribution of active sources in the brain, which means that the relative contribution, location, or orientation of at least some of the sources differ, and a different spatial configuration of the scalp field is observed (case B1 in Figure 6).

Data normalization eliminates all differences between the maps which are solely due to scaling of the entire scalp fields. Thus, this scaling factor may differ between conditions, but is, within each condition, the same for all channels. Technically, data normalization is achieved by dividing all potential values of a given map by its GFP.

If the data was normalized before performing further tests such as topographic analysis of variance (TANOVA), significant differences found between the conditions can be attributed to changes in the spatial distribution of the active sources in the brain. This would indicate a change in the relative spatial distribution, location, or orientation of the active sources between the conditions. This is typically interpreted as a change in mental strategies. Significant scalp field differences encountered after normalization are thus sometimes referred to as *qualitative* differences.

The analysis of normalized data can and should be complemented by analysis of the scaling factor, which is the GFP in this case. In Ragu, this analysis is implemented in a form parallel to the TANOVA and available in the Analysis menu as “GFP/RMS.” If the analysis of the GFP yields significant results in the absence of topographic effects, it is sometimes referred to as a *quantitative* effect. TANOVAs without prior normalization tend to yield overall results that may contain a mixture of qualitative and quantitative effects. Normalizing the data before computing the TANOVA, and performing additional analysis on the GFP will disentangle these two types of effects.

#### 4.1.2. Randomization runs

The number of randomization runs defines the number of times a chosen test is repeated with shuffled data in randomization statistics. If more randomization runs are performed, the obtained probability distribution under the null hypothesis becomes more accurate. Referring to the potato example, this option would define the number of times the potato samples from the two farmers are mixed and evaluated. Five thousand randomization runs are publication standard, but one thousand runs are the recommended number for an accurate estimate of significance at the 5% level (Manly, 2007). Note that as the number of randomization runs increases, the computation time increases linearly and may eventually make the analysis of larger datasets time-consuming. For purely exploratory analyses, it is thus often useful to substantially lower the number of randomization runs to save computation time.

#### 4.1.3. P-threshold

The rejection of the null-hypothesis based on its probability requires that the *p*-value reaches a critical lower threshold. The failure of *p*-values to reach this threshold will then be considered as a reason to accept the null-hypothesis. The choice of the p-threshold thus determines how liberal or conservative the significance testing of an analysis will be. In Ragu, the chosen p-threshold is used in all the tests based on randomization statistics. This chosen p-threshold determines which results are marked as significant in some of the displays and plays a role in the performance of some of the overall statistical analyses (see section 4.3.1).

### 4.2. Topographic consistency test

A standard assumption in the group analysis of ERPs is that within a defined experimental group, the subjects activate, at least partially, common processing resources. In neurophysiological terms, this translates into the assumption that the event elicits the activation of a common set of sources. But while this assumption seems to be essential for most of the conclusions typically drawn in group analyses, it is rarely evaluated. Additionally, apart from validating some basic assumption, such a test may facilitate to empirically establish the analysis time window as the period where this assumption factually holds.

The *topographic consistency test* (TCT) will help us to determine if, for a given moment or period of time, an experimental condition elicited a consistent neural activation across subjects. In our example, we want to know when the subjects reacted similarly to the word stimuli in a particular condition in terms of ERPs. If such consistency across subjects cannot be found in the data, it is impossible to attribute ERP reactions to an experimental manipulation, since there is no evidence that the conditions elicited ERPs in a verifiable way. To determine if there is evidence, for a given time and condition, that there is a set of sources sufficiently common across subjects to be detected on the level of the grand-mean, the TCT is the method of choice.

How does this test work? First, a quantifier that is sensitive to the spatial consistency of ERP maps across subjects has to be specified. This can be found in the GFP of the grand-mean map across those subjects, because the GFP of the grand-mean ERP map (mean map across all subjects at one time point) depends not only on the amplitude of the individual maps, but also on the spatial consistency of these maps across subjects.

**Figure 7:**
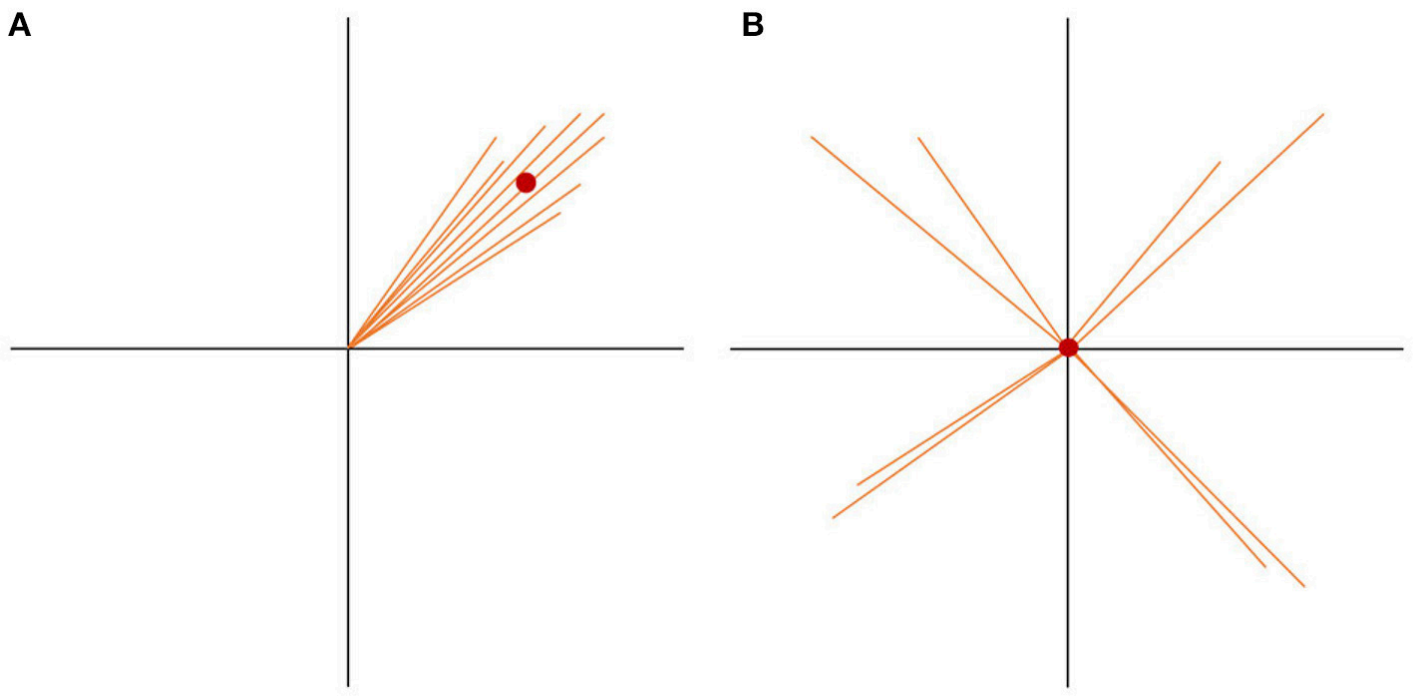
State-space representation of the randomization statistics used for the TCT. In order to visualize the argument, only two channels (E1, E2) are shown. **(A)** Shows the distribution of data with high consistency. The vectors represent the individual maps. The red dot is the grand-mean map, which has a relatively large GFP. **(B)** Shows a random distribution of the data. Note that only the orientation of the individual data, but not the length, has been randomized. Thus, the GFP of each vector stays the same. Notice how the grand-mean map moves to the origin in the case of the random data distribution. It becomes obvious that the GFP of the mean ERP map depends not only on the GFP of the individual maps, but also on the spatial consistency across the individual data. This is the reason the GFP of the mean ERP map of one condition can be chosen as a measure of effect size for consistency.

If there are more spatially consistent activities across subjects, the GFP of the grand-mean map becomes large (see Figure 7A). If there are a lot of differences in the individual maps, the potential values get canceled out during the computation of the grand-mean map, and consequently, the GFP of the grand-mean map is small (see Figure 7B). We can therefore state that the GFP of the grand-mean ERP map depends systematically on the consistency of active sources across all subjects.

To test if the obtained GFP could be produced by ERP maps that have no consistency across subjects, we need data which reflects the null hypothesis. The null hypothesis states that consistency between subjects is produced by chance and should be relatively small. To produce this random data, the structure of every map can be “destroyed” by shuffling separately for each subject, the measured potentials of each map across channels. By doing so, the mean GFP of each subject remains the same, but the potentials are randomly distributed over the channels. The grand-mean map of this random data is then computed across subjects, followed by the computation of the GFP of this random grand-mean map. The obtained GFP value is thus an instance of a GFP value that is compatible with the GFP values of all given individual maps in the absence of any systematic communality of these maps across subjects. This corresponds to our null-hypothesis.

The shuffling of the data and the computation of the grand-mean GFP is then repeated multiple times, in order to obtain a distribution of the probability of the grand-mean GFP under the null hypothesis. Finally, the probability of the observed GFP can be tested by computing the percentage of cases where the GFP obtained after randomization is equal to or larger than the observed GFP. The TCT, from a set of ERPs recorded in different subjects but in the same condition, produces a time-series of *p*-values that indicate the probability that the GFP of the grand-mean ERP across subjects is compatible with the null-hypothesis.

**Figure 8:**
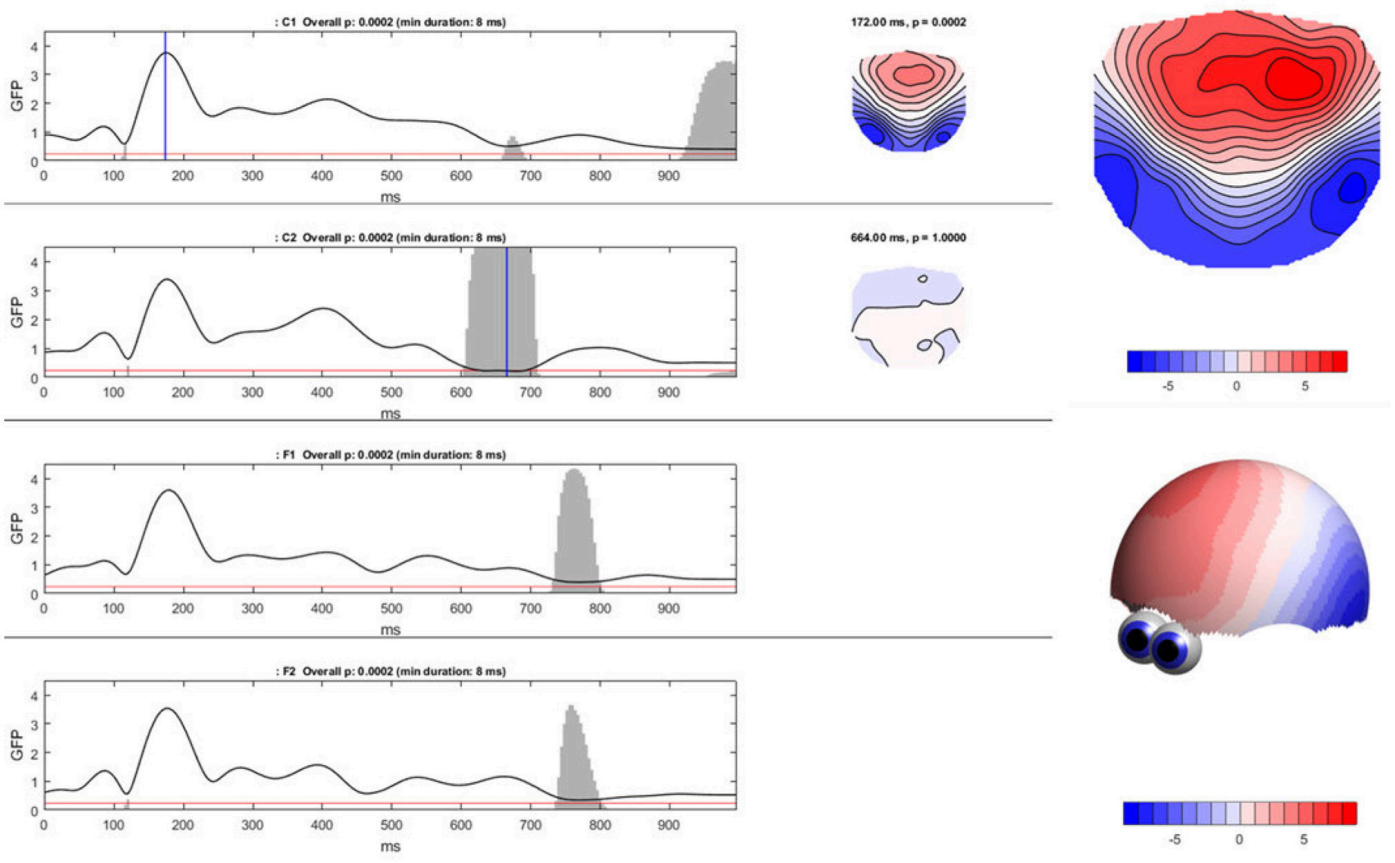
Ragu output for the TCT. **Left:** The Global Field Power (shown as the black line) of the mean ERP maps on the y-axis for every time point in ms on the x-axis is shown separately for each condition. The red line indicates the p-threshold (0.05). The gray area marks non-significant time points. The height of the gray area indicates the *p*-value of the TCT (in the white area *p* < 0.05). **Right:** By clicking on the graph, the mean ERP map of a specific time point can be shown, as displayed on the right side of the GFP curves. There is a clear contrast between the moments of high GFP (in the first graph; C1) and small GFP (second graph; C2) displayed by a more intense coloring and narrower contour lines. It is possible to compute t-maps and plot three-dimensional models in this dialog window (see picture on the right).

To compute the TCT in Ragu, click “Analyses/Results→Topographic Consistency Test.” The resulting graph indicates that—as expected—for all conditions and most of the analysis period, the GFP of the grand-mean ERP map across all subjects is larger than it would be by chance. There is therefore evidence for a significant communality across subjects. However, the results become additionally informative when comparing the conditions (see Figure 8). We observed an earlier onset of a non-significant time period indicating the end of an identifiable “cognitive rule” in C2, as compared to the C1 and F-conditions. Thus, in the F-conditions and in C1, the relevant effect of the stimulus seems to last longer than in the C2-condition. There might be an effect of time between C1 and C2 which could reflect either a learning or interaction effect. However, to test this, further steps for analysis are required because we only have information about the consistency within the conditions, but no information about potential significance of differences or relationships between the conditions.

### 4.3. Topographic analysis of variance

The TCT showed that there was consistent neural activity in the conditions across all subjects most of the time. The next question that arises is if there are significant map differences between the expected and the unexpected sentence endings and/or between day 1 and day 2. To compare map differences between factor levels, a *topographic analysis of variance* (TANOVA) can be performed. The TANOVA tests for significant differences in the maps between factor levels. This is essentially done by quantifying the strength (i.e., the GFP) of the difference maps between those factor levels. These difference maps are interesting for us because they indicate if different sources were active at the specific factor levels in our experimental design. By analyzing the difference maps, we can draw conclusions about our hypotheses and the success of our experimental manipulation. The difference maps represent the actual physiological outcome of our experimental manipulation.

TANOVA works by averaging the potential maps for each factor level separately, followed by computing the difference maps between factor levels. This can be performed for each factor, and for their interactions. The GFP of the difference maps can then be taken as a global quantifier for the differences between all factor levels (see Equation 2, Michel et al., 2009, p. 177). To ensure that the differences are not just produced by chance, randomization statistics are applied. If there is the need for exploratory data analysis, TANOVAs can be computed for each time-point, eventually followed by corrections for multiple testing across time (see section 4.3.1). If there is a specific time-window of interest, the TANOVA can also be used for producing scalp maps averaged across this time window.

(2) $$\begin{array}{l} dGFP=\sqrt{\frac{\overset{c}{\underset{i=1}{\sum }}\overset{n}{\underset{j=1}{\sum }}{\left({\overset{-}{v}}_{ij}-{\overset{-}{\overset{-}{v}}}_{j}\right)}^{2}}{n}}, \end{array}$$

where *c* is the number of factor levels (or combinations thereof, if an interaction is to be tested), *n* is the number of channels, ${\overset{-}{v}}_{ij}$ is the grand-mean across subjects of the voltage of factor level *i* at channel *j*, and ${\overset{-}{\overset{-}{v}}}_{j}$ is the grand-mean across subjects and factor levels of the voltage at channel *j*. All data are recomputed against the average reference.

**Figure 9:**
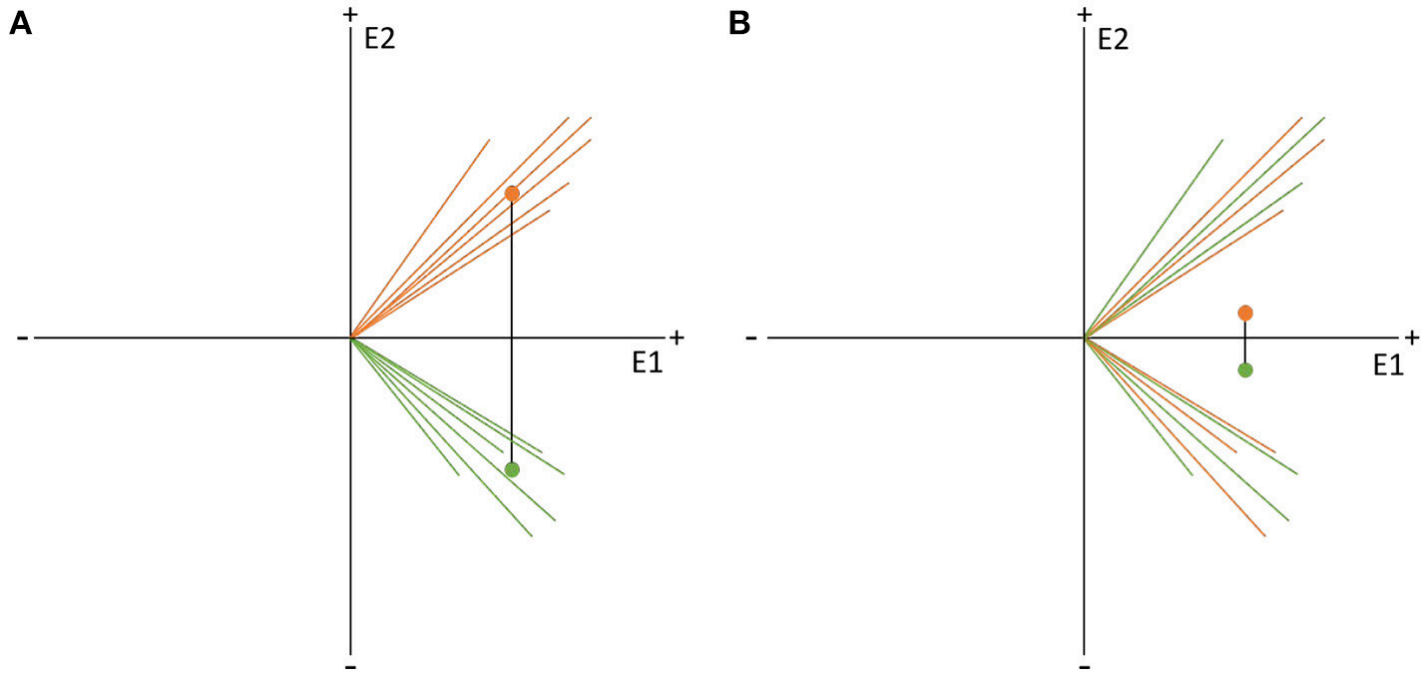
Visual representation of the data randomization used to compute a TANOVA. The two colors indicate the data resulting from two different factor levels, e.g., the expected and unexpected sentence endings on the two channels, E1 and E2. The dots represent the means of the factor levels. The length of the black line between the two means indicates the strength of the difference between the factor levels which is equal to the GFP of the difference map. **(A)** Shows an example of very different factor levels. **(B)** Shows the same data after randomization between the two factor levels across subjects. Notice that the difference becomes quite small when the data is randomized. This comparison indicates that randomized data should produce difference maps with with relatively small GFP values.

Specifically, to sample GFP values of difference maps (dGFP, see Equation 2) under the null hypothesis, factor levels are shuffled within subjects, and the computation of dGFP is repeated (see graphs A and B in Figure 9). This step is repeated multiple times to obtain a distribution of probability for dGFP under the null hypothesis (see section 4.1.2). The observed dGFP of a factor or interaction can thus be evaluated for significance by comparing it with the distribution of dGFP values under the null hypothesis. The probability of the null hypothesis is then defined as the percentage of cases where the dGFP values obtained after randomization are equal to or larger than the dGFP value obtained in the observed data.

**Figure 10:**
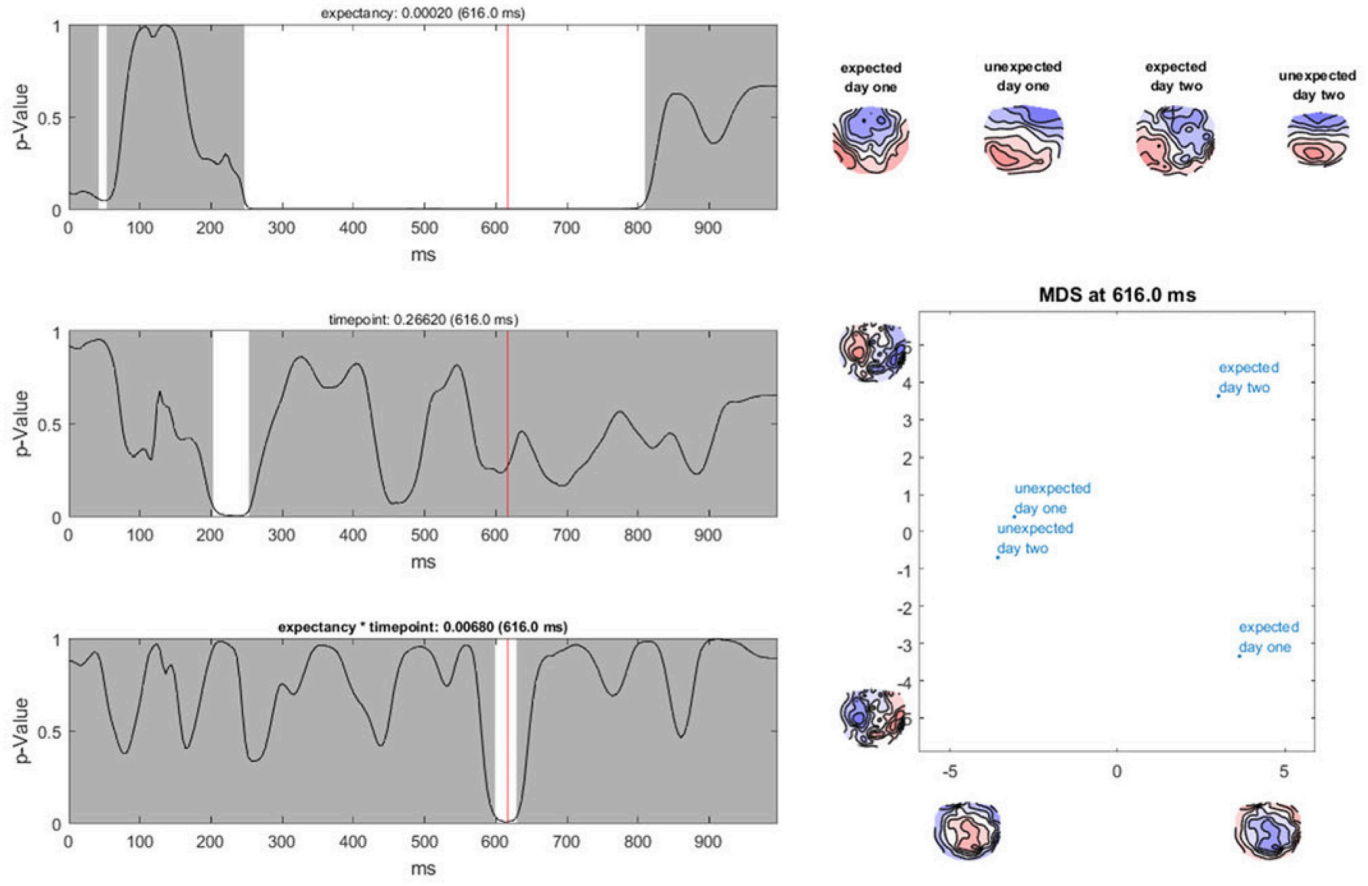
TANOVA results. **Left:** *P*-values (y-axis) for the comparison between the mean ERP maps of each factor level and the interaction for every time point in ms (x-axis). The gray areas mark non-significant time points while the white areas mark periods of significant differences between ERP maps of different factor levels. **Right:** the four-level interaction (combination of the two main factors) is shown at a significant time point combined in a graph using a state-space representation in order to display the relationships between the factor levels. In this figure, the ERP maps of every single factor level producing an effect are displayed.

Ragu displays the result of a TANOVA in a set of graphs (Figure 10). The first set of these graphs shows, for all factors and their combination, the obtained *p*-values as a function of time (Figure 10, left). Clicking on a graph allows the user to select a particular time point and factor or interaction for further visualization (Figure 10, right). In our case, the display shows the *p*-value of the factors “expectancy” and “day” and the 2 × 2 interaction of the two factors over the entire time course. The right side of the display further disentangles the interaction of the factors at the chosen time point (616 ms).

In the upper right part of this display, the mean maps across subjects of all factor levels that form the chosen effect are shown. To display the relationship between these maps, the state-space approach is used. In the introduction (section 3.1), we learned that a map can be considered as a vector in an n-dimensional space, where n is the number of channels. Differences within a given set of maps are then represented as differences among those vectors. To optimally visualize map differences on a two-dimensional computer screen, this n-dimensional space is rotated until its projection on a two-dimensional surface is maximally informative about the relationships among all maps to be represented. For this purpose, a *principal component analysis* (PCA) is computed based on all these maps. The eigenvalues of the first two principal components are displayed on the x- and y-axes of a scatter plot (Figure 10, lower right corner). Additionally, the PCA-eigenvector-maps are shown on the x- and y-axes. The location of the points represents different maps. Conclusions about the relationship between these points can be drawn. If the dots representing two mean maps are close, the maps are considered to be relatively similar, and if the dots are further away, the maps are considered to be different. We can see that in the display, the maps elicited by expected sentence endings differ much more in location compared to those elicited by unexpected sentence endings. The maps on the axes of the graph inform us that this shift in locations is associated with a negativation, predominantly over the midline, central, and parietal regions for expected sentence endings.

In our sample analysis, there is a time period between 250 and 800 ms after the stimulus onset, in which the maps of the correct and false sentence endings were significantly different from one another. For the factor “day,” there is only a small window of significance. In addition, for the interaction effect between the factors “expectancy” and “day,” there is only a short period of significant dissimilarity between all factor levels. This suggests that if the sentence ended as expected, different neural processes were activated during the period between 250 and 800 ms after stimulus onset compared to the cases when the sentence ended in an unexpected way. Furthermore, there seems to be an interaction between the factors “expectancy” and “day” for a neural activation which takes place between 580 and 640 ms after stimulus onset. The state-space display suggests that this interaction is observed because the maps for the false sentence endings do not vary much from day 1 to day 2, but the maps for the correct sentence endings change. This could reflect a learning process for correct German words we already hypothesized after computing the TCT. To investigate this theory more precisely, *post-hoc* tests are required. However, before we describe such, we need to address the issue of multiple testing in time, which is referred to as “Overall Statistics” in Ragu.

#### 4.3.1. TANOVA overall statistics

It is obvious that applying time-point by time-point TANOVAs, as we did in the previous section, creates a problem of multiple testing. This problem is confounded by the fact that among the time-points, there is an a-priori unknown dependence, such that a correction by the full number of tests conducted would be overly conservative. It is, however, unclear what the proper correction factor should be. Ragu has a number of solutions to this problem. It allows correction for multiple tests that are to an unknown degree interdependent, such as our TANOVA results. Note however that these approaches may still be overly conservative in light of pre-existing knowledge about the functional correlates of certain analysis periods.

In Ragu, the options to correct for multiple testing over time are called overall statistics. These options become available after the computation of the TANOVA (Analyses/Results→Tanova Overall Stats).

What is the principle behind these overall statistics? In the TANOVA, a test for significance can be computed for every time point. This yields a distribution of *p*-values, of which a subset eventually ends up being below a chosen significance threshold by chance alone, and thus constitute false positives. To minimize this problem, we need to test the obtained distribution of *p*-values against a distribution of *p*-values that is compatible with the null hypothesis. Ragu allows these tests to be performed for three quantifiers of the distribution of *p*-values, namely the amount of *p*-values below a certain threshold, the duration of contiguous periods with sub-threshold *p*-values, and a combination of all obtained *p*-values. We will now illustrate this in more detail for the case of the amount of sub-threshold *p*-values.

**Figure 11:**
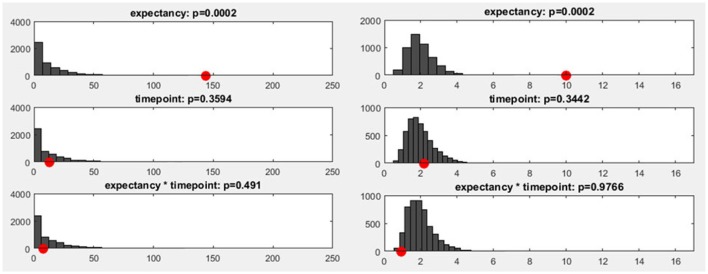
**Left:** The y-axis shows the number of randomization runs in which a certain number of sub-threshold time points was obtained. On the x-axis, the sampling points in the data are displayed (time: 1,000 ms, sampling rate: 250 Hz, 250 sampling points). The red dot marks the count of sub-threshold time points obtained in the observed data. The dark gray bars mark the amount of randomization runs in which a certain number of sub-threshold time points were obtained with the shuffled data. **Right:** The distribution of the meta-analyzed results obtained using Fisher's method is shown. The gray bars indicate the results from the randomized data while the red dot marks the observed effect size.

To estimate how likely it is that a certain number of sub-threshold *p*-values are present under the null hypothesis, we can employ a procedure called “Global Count Statistics.” The procedure re-uses previously obtained results of randomization procedures: For the TANOVA, we have already computed GFP values of difference maps among factor levels, both for the original data and after randomizing the assignment to the experimental conditions. These GFP values can now be further used for the overall significance tests. For every randomization run computed during the TANOVA, *p*-values can be computed by comparing the obtained random differences with those obtained in all other randomization runs. This allows us, in the next step, to extract the number of sub-threshold *p*-values that we can expect when the null-hypothesis holds. The result is thus an estimate of the distribution of the count of false-positives under the null-hypothesis. Finally, the count of sub-threshold *p*-values in the observed data can be compared to this distribution of the count of false positives in the random data. Given that we chose a 5% p-threshold for the overall significance, the output of the overall significance test then indicates a count that is larger than 95% of the false positive count obtained in the random data (see Figure 11), and thus expectedly produces an overall 5% false positive rate.

**Figure 12:**
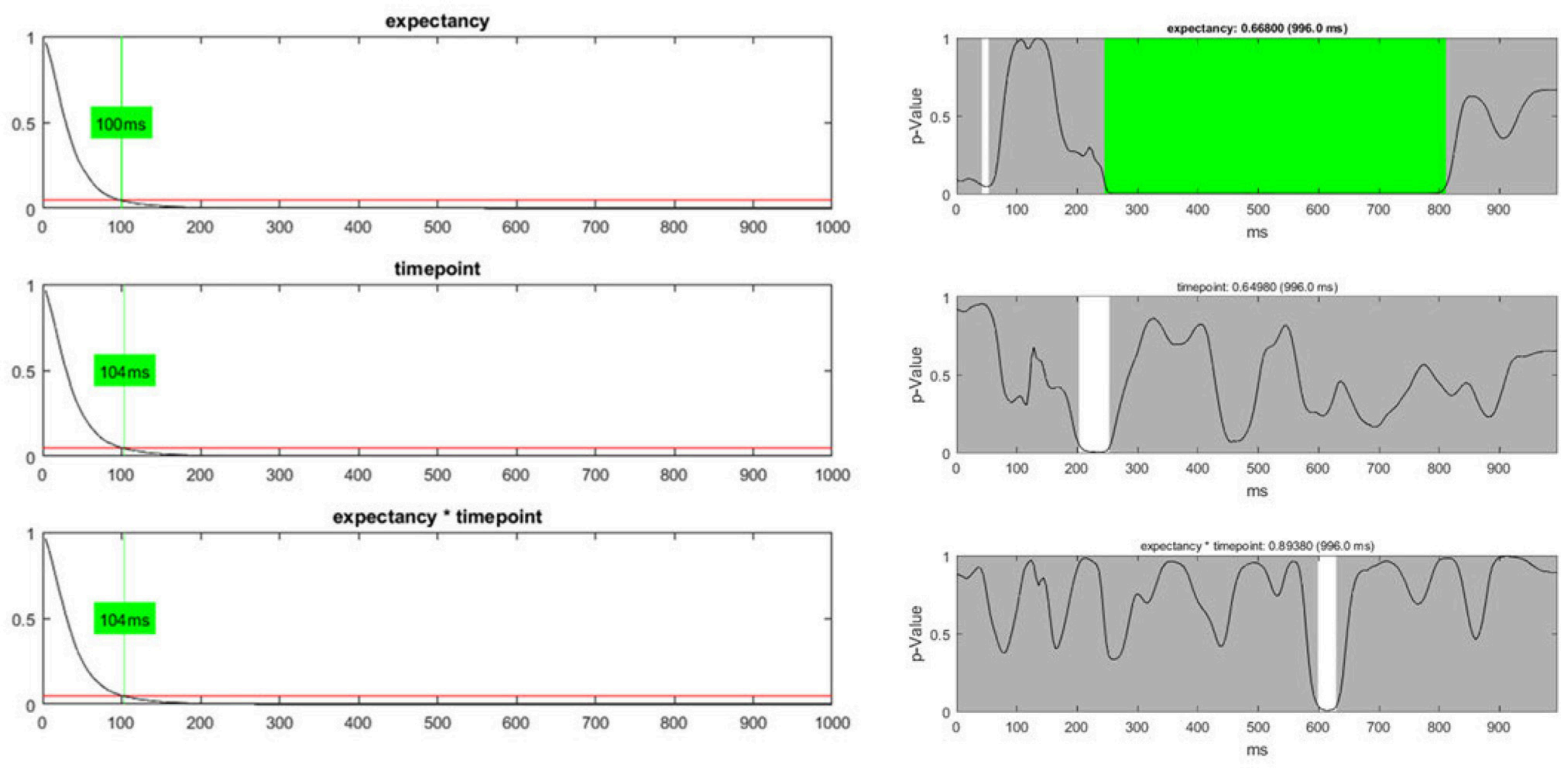
**Left:** The global duration statistics for the different factors are shown. The y-axis indicates the *p*-value; the red line represents the p-threshold (5%). The x-axis represents duration in ms. The curves shown are the distribution of the length of continuous periods with sub-threshold *p*-values that can be expected under the null hypothesis. The green line and the indicated duration mark the length of succeeding sub-threshold time points in the randomized data which is less likely than the chosen significance level. These duration thresholds are then applied to the TANOVA plots, where periods longer than the estimated duration threshold are marked in green. The only factor for which the observed length of sub-threshold time points is significant is the “expectancy” factor (see the first row). **Right:** The green area on the right-hand side identifies periods in the TANOVA that meet the extracted duration thresholds (green areas).

In addition to the count of false positives, it is also possible to combine the set of all obtained *p*-values (“Global p-AUC Statistics”) using Fisher's method (Fisher, 1925). The resulting summary values are then tested for significance using the same logic as the one employed for the duration and count of significant time points. Finally, one can perform the same type of analysis for the duration of continuous periods with sub-threshold *p*-values (“Global Duration Statistics”). As output, one obtains a value for the duration of periods with sub-threshold *p*-values that needs to be exceeded if an effect will be considered significant in the overall analysis (Figure 12).

In our dataset, only the “expectancy” factor survives any of the overall statistics. However, the overall statistics, as presented and applied here, assumes a situation where one is completely naive about the outcome of our study and has no other information but the data. This is typically not the case. Usually, one starts the experiments with some reasonably founded expectations about, at least, parts of the outcome, and may take this background information into account when weighing the evidence obtained from the data. Thus, the overall statistics may sometimes end up being overly conservative in light of previous knowledge and may also be treated as such. As this paper aims at illustrating typical analysis strategies using Ragu, and not at advancing our empirical knowledge about language learning, we will assume that we have additional background information that makes us reasonably expect a late interaction effect. We thus continue the analysis using *post-hoc* tests in order to specify this interaction effect, with the sole aim of demonstrating how such interactions can be performed.

#### 4.3.2. *Post-hoc* tests (t-maps)

The TANOVA revealed a period of sub-threshold (<0.05) *p*-values for the interaction effect between the factors, “expectancy” and “day.” While this interaction did not survive the overall statistics, we assume that based on the literature, we have reasons to believe that the time range of the identified interaction is compatible with existing knowledge about language, semantic integration, and learning, and thus merits attention nevertheless. However, at the current state of analysis, the output of the TANOVA is very general and does not indicate specifically which factor levels primarily account for the effect, and what the topographical characteristics of the effect were. *Post-hoc* tests and t-maps will help us to access this kind of information.

In Ragu, *post-hoc* tests and t-maps are computed for a specific contrast of interest, and for the average over a user-defined time period. The dialog window for computing t-maps is accessed through the menu (Analyses/Results→t-Maps & sLoreta). The dialog window permits the user to select specific subject groups and conditions to be contrasted, and define a time window for the averaging across time. Eventual baseline conditions may also be taken into account. The test type (paired vs. unpaired), is set automatically.

In our example, we search for specific information about the time interval in which the TANOVA showed an interaction effect, i.e., between 580 and 640 ms after the stimulus onset. T-maps indicate, for each channel, how much some conditions differ on the average, in comparison with the variance of the differences across observations, and thereby provide a local index of the signal-to-noise ratio on the scalp. T-maps contain information that an educated viewer may use to make some preliminary inferences about the location of the active sources that produced the differences in the conditions, like the gradients and poles of the map. Note, however, that due to volume conduction, the scalp location of large *t*-values does not necessarily coincide with the location of the brain sources that account for these differences (see also Figure 1). In our example, it is particularly interesting to analyze the time period with the interaction effect.

**Figure 13:**
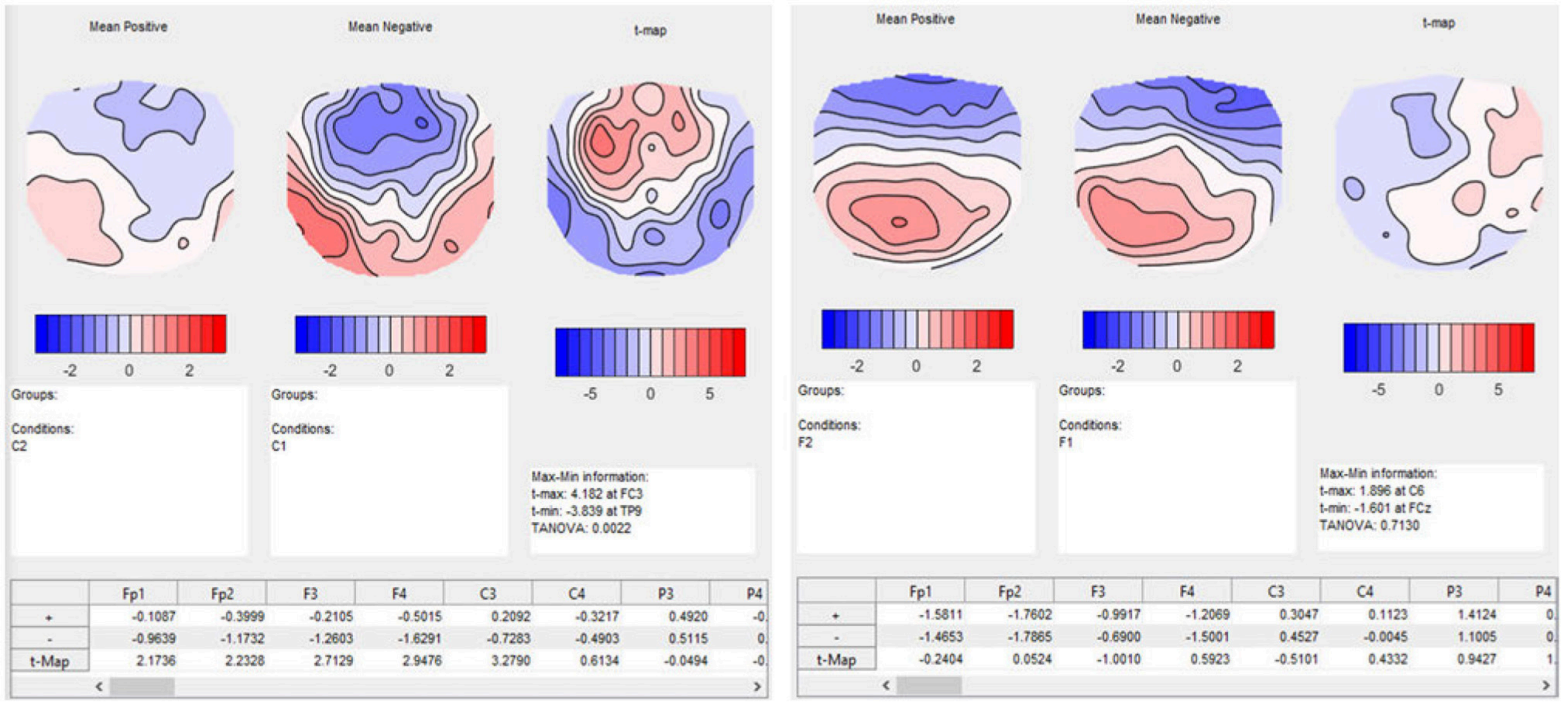
Results of t-mapping. **Left:** the ERP mean maps for the expected sentence endings are displayed for day 1 and 2. The resulting t-map is also shown. Day 1 was subtracted from day 2 to parallel the temporal sequence of the conditions, and thus the learning effect. The dialog window also provides further information, including information on the *p*-value of the TANOVA, and a table with the *t*-values for each channel. **Right:** The ERP maps of day 1 and 2, and the corresponding t-map, are shown for the unexpected sentence endings. The t-map for unexpected sentence endings is much flatter than that for the expected sentence endings, and the TANOVA is not significant.

In our *post-hoc* analysis for the interaction effect, we test a series of differences between the conditions in the time period between 580 and 640 ms after the stimulus onset. The results of the TANOVA already suggested that there is a relatively large difference between the conditions C1, expected sentence ending on day 1, and C2, expected sentence ending on day 2, while the difference between the F-conditions, i.e., the unexpected sentence endings, is much smaller. A paired t-map, combined with a TANOVA, quantifies the potential learning effect between C1 and C2 in isolation. The resulting t-map can then be compared to the t-map of the F-conditions. As expected, the t-map of the C-conditions shows larger values for the comparison of day 1 and 2 than the t-map of the F-conditions (Figure 13).

As a result, we obtain the t-maps of the C- and F-condition for day 1 and 2 (see Figure 13). The output also contains a table with the *t*-values for each channel. We investigated the time period of the interaction effect that we found in the TANOVA. The values of the t-map for the comparison between the expected sentence endings are much larger than the values of the t-map of the unexpected sentence endings. As expected, the significance values from the TANOVAs shown below the t-maps are also quite different. While the map of the correct sentence endings changed considerably between day 1 and day 2 *(p* = *0.0022)*, the maps for the false sentence ending remained nearly the same *(p* = *0.7130)*. This is evidence for a selective learning effect in the perception of expected sentence endings, but not in that for the unexpected sentence endings.

### 4.4. Microstates analysis

The TANOVA allowed us to test whether the different experimental conditions elicited different brain functional states at a given time point, and provided information which proved to be informative. However, no information was provided concerning the latency differences of particular components. Asking about latency differences in ERP components is similar to asking if, for some defined brain functional states, their observed presence varies systematically as a function of our experimental design. Microstate analyses can help us answer this type of questions. Microstate analysis comprises the examination of brain electromagnetic scalp data in terms of a set of fixed maps, and quantifying the data by the time periods (i.e., microstates) when these maps are predominant (Brandeis et al., 1995). Technically, microstate analyses belong to the broad family of spatial factorization procedures, among which spatial-principal component analysis and mainly the independent component analysis (Makeig et al., 1997) are also important members. All of these procedures decompose a multichannel EEG or ERP time series into a time-varying linear combination of a relatively small set of spatial maps or components, while optimizing this decomposition for certain additional a-priori objectives of independence among the time-courses of components: While spatial principal component analysis aims at linear independence, independent component analysis eliminates also higher order dependencies. Finally, microstate analysis implements independence by excluding any temporal overlap of components, such that there is, for a given time, only one active component (Pascual-Marqui et al., 1995). While the rationale of any of these procedures is certainly debatable, microstate analysis has proven sufficiently to have some justification. A relatively small set of microstates is typically enough to account for a large part of the data, and their quantification often yields results that correspond well with theories that predict e.g., reaction time differences (see e.g., Schiller et al., 2016).

Technically, microstate analysis typically consists of a clustering step, where the scalp field data to be analyzed is submitted to a spatial clustering algorithm that identifies the component/microstate maps, and an assignment step, where the individual time-points and conditions of the data are assigned to the best-fitting cluster, yielding the time-courses of the microstate model (Pascual-Marqui et al., 1995). In Ragu, both of these steps are typically applied to factor level- and group wise averaged grand-mean evoked potential data. A microstate analysis thus yields two types of information. First, there is, for a given dataset, a set of scalp maps that represent the cluster centers, or microstate maps. Second, there is an assignment, for each group and factor level, of each moment in time to one of these microstate maps. This assignment becomes the basis for the subsequent statistical analyses, where a microstate is defined as a continuous period of time that has been assigned to the same microstate map. Different quantifiers of these microstates can then be extracted and statistically tested (see below). Apart from the fact that microstate analysis yields temporally sharp and unequivocal information about the onset and offset of each component, the fact that empirically, microstates often cover extended time periods suggests that there is, within each microstate, a predominant synchronization of the contributing sources (Michel and Koenig, 2017).

As different microstates represent different activations from underlying neural sources, it can be argued that they reflect different types of mental processes (Khanna et al., 2015). Microstate analysis can be used to investigate if certain brain processes differ in their timing between factor levels, i.e., if their length, onset, or offset latency was systematically affected by the experimental manipulation. For the demonstration of microstate analyses in Ragu in our sample data, we will focus on the following two components:

- **N400 and ERP microstates**: Previous studies showed that if the semantic expectancy of a subject is violated, an ERP effect is produced about 400 ms after the stimulus onset (Kutas and Hillyard, 1980). This ERP component is called N400 and it is accompanied by a negativity over central scalp sites (Brandeis et al., 1995). In our study, we would therefore expect a corresponding difference in the microstate maps between the expected and unexpected sentence endings, about 400 ms after stimulus onset. The unexpected sentence endings should be marked by a microstate which has a central/parietal negativity. This hypothesis is supported by previous findings from Brandeis et al. (1995). In this study, the ERP microstates corresponding to expected or unexpected sentence endings were mapped and a map with posterior negativity around the N400 was found if the sentence ending was unexpected.
- **Interaction effect**: In the TANOVA, we found a 2 × 2 interaction between the factors “expectancy” and “day” between 580 and 640 ms after stimulus onset. Now we want to specify this effect using microstates, because there may be diverging ways to account for the differences between the ERP maps we observed in the TANOVA. One possibility is that very similar mental processes were activated in all the conditions, but they had different durations. A dissimilarity between the conditions would then be due to a delay in one condition compared to another. Alternatively, different mental processes may have been active in the different conditions, but with a similar timing, which also would yield an effect in the TANOVA. Microstate analyses can help to distinguish these possibilities.

#### 4.4.1. Number of microstate classes

At the beginning of any microstates analysis, the number of microstate classes has to be determined in a meaningful way. This can be done a-priori, and based on previous studies, or empirically by cross-validation (Koenig et al., 2014). The cross-validation procedure aims to identify the number of microstate maps that are optimally predictive for new data. For this purpose, the subjects of the dataset are randomly divided into a learning set and a test set. For our example, we will define a learning set and a test set each containing 50% of the subjects. Next, the learning set is used to construct a set of spatio-temporal microstates models that vary in the number of microstate classes. Each of these spatio-temporal models consists of a set of maps and an index that assigns each moment of time of the data to one of these maps. This spatio-temporal model is then projected onto the test set, which yields, for each moment in time, the amount of variance of the test set explained by the microstate model obtained in the learning set. Finally, the overall explained variance across time is computed for the test set. The models are then evaluated based on the explained variance. As expected, in the learning set, the explained variance will increase with every microstate class added. Yet, in the test set, the explained variance will stop increasing after a certain number of classes, which is an indicator of the beginning of over-fitting the data. In a graph, this should be observed as a plateau in the curve of explained variance. The best fitting number of microstate classes for the data is marked by the beginning of this plateau, because adding more microstate classes beyond this point does not add generalizable features to the model.

#### 4.4.2. Randomization statistics

Once the number of microstate maps has been set, randomization statistics can be computed for the assignment of every microstate class and for all factors and their interactions. The goal of microstate statistics in Ragu is to compare particular microstates and their assignment between the factor levels, and to test eventual differences between groups and factor levels for statistical significance. Ragu does this by extracting different quantifiers for each microstate class. They include the onset latency, offset latency, duration, area under the curve (AUC), center of gravity^4^, and the mean GFP. The duration, AUC, and mean GFP of the microstates are global measurements of the occurrence of particular microstates, whereas the onset, offset, and center of gravity provide more information about the behavior of the microstates in time. Among the temporal parameters, the center of gravity is the most robust parameter, because it depends on all data points and not solely on the first and last observations of the microstate class.

The randomization statistics for the microstate analysis work as follows: First, the quantifiers described above are computed in the grand-mean ERP maps for every factor level and group, and the variance between factor levels and groups is extracted. Next, individual ERPs are assigned to individually shuffled factor levels, and group labels are also randomly shuffled. New grand-means are then computed based on these shuffled individual ERPs. The same quantifiers are extracted and their variance is computed. By repeating these shuffling, quantification, and comparison steps for a number of times, one can obtain a distribution of the variance between factor levels and groups of each of these quantifiers under the null-hypothesis. The significance of the observed variance between group and factor level means can then be estimated based on the obtained random distributions (Koenig et al., 2014).

#### 4.4.3. Computing and testing microstates in ragu

**Figure 14:**
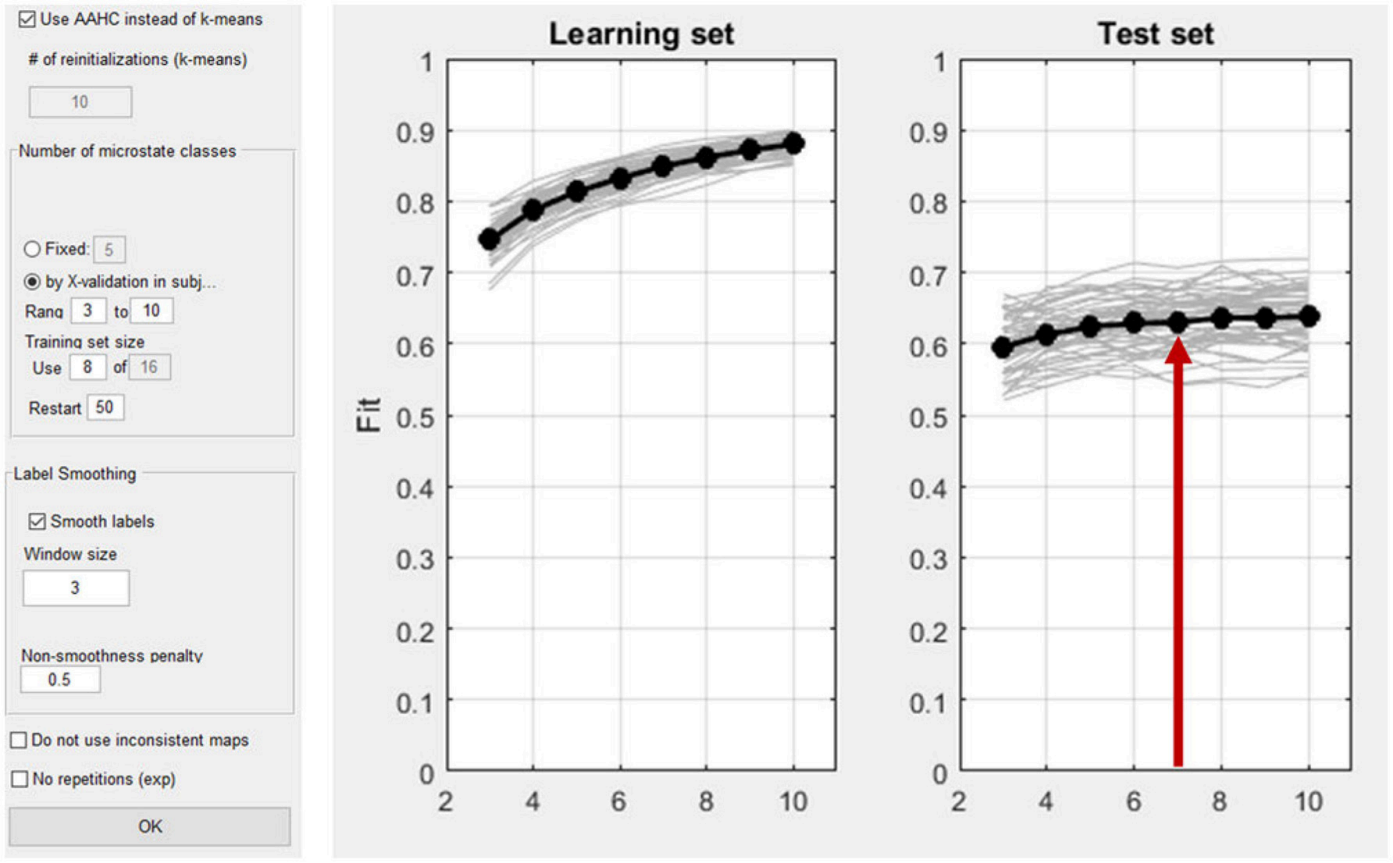
**Left:** Dialog window for the computation of microstates in Ragu. **Right:** The result of the cross validation for the optimization of the number of microstate classes. The x-axis shows the number of classes of the different solutions (3–10). On the y-axis, the explained variance (the fit) is displayed. The left plot shows the explained variance in the learning set, which by nature of the analysis increases monotonically. The left plot shows the explained variance in the test set, which stops increasing after a certain number of microstate classes. The red arrow marks the point in the test set where a plateau of explained variance seems to be reached. The best fitting model seems to be the seven-microstate class solution. However, it may be reasonable to also explore other numbers to ensure that the results do not crucially depend on that particular choice.

As explained above, first the microstate maps have to be computed (Analyses/Results→Microstates→Compute Microstate maps). The time interval of interest can be defined. In our example, the whole time window is analyzed. As shown in Figure 14, the microstate dialog window permits the user to choose a cluster-algorithm. Either the *atomize and agglomerate hierarchical clustering* (AAHC) algorithm (Murray et al., 2008) or the *k-means* algorithm (Pascual-Marqui et al., 1995) can be chosen. The number of microstate classes can be determined either by a-priori assumptions (the user should then select “fixed”) or alternatively using cross validation (Koenig et al., 2014). In our example, cross-validation is performed by testing a range from 3 to 10 microstate classes. If the option “Smooth labels” is checked, very short microstates are suppressed (Pascual-Marqui et al., 1995).

After the identification of an appropriate number of microstate classes (see Figure 14, right), the next step is to compare the microstate assignment and the resulting parameters between the factor levels. To do so, randomization statistics are computed (Analyses/Results→Microstates→Microstate Fitting Statistics) as described earlier.

#### 4.4.4. Interpretation

As there is a wealth of information resulting from this analysis, we focus on two components that we planned to investigate originally. We had specific hypotheses about microstates corresponding to the N400 effect, and to the interaction effect in the time window 580 and 640 ms after stimulus onset. There may be more information in the data, but rather than becoming speculative, we demonstrate the interpretation of results within the frame of our hypotheses.

##### 4.4.4.1. N400

**Figure 15:**
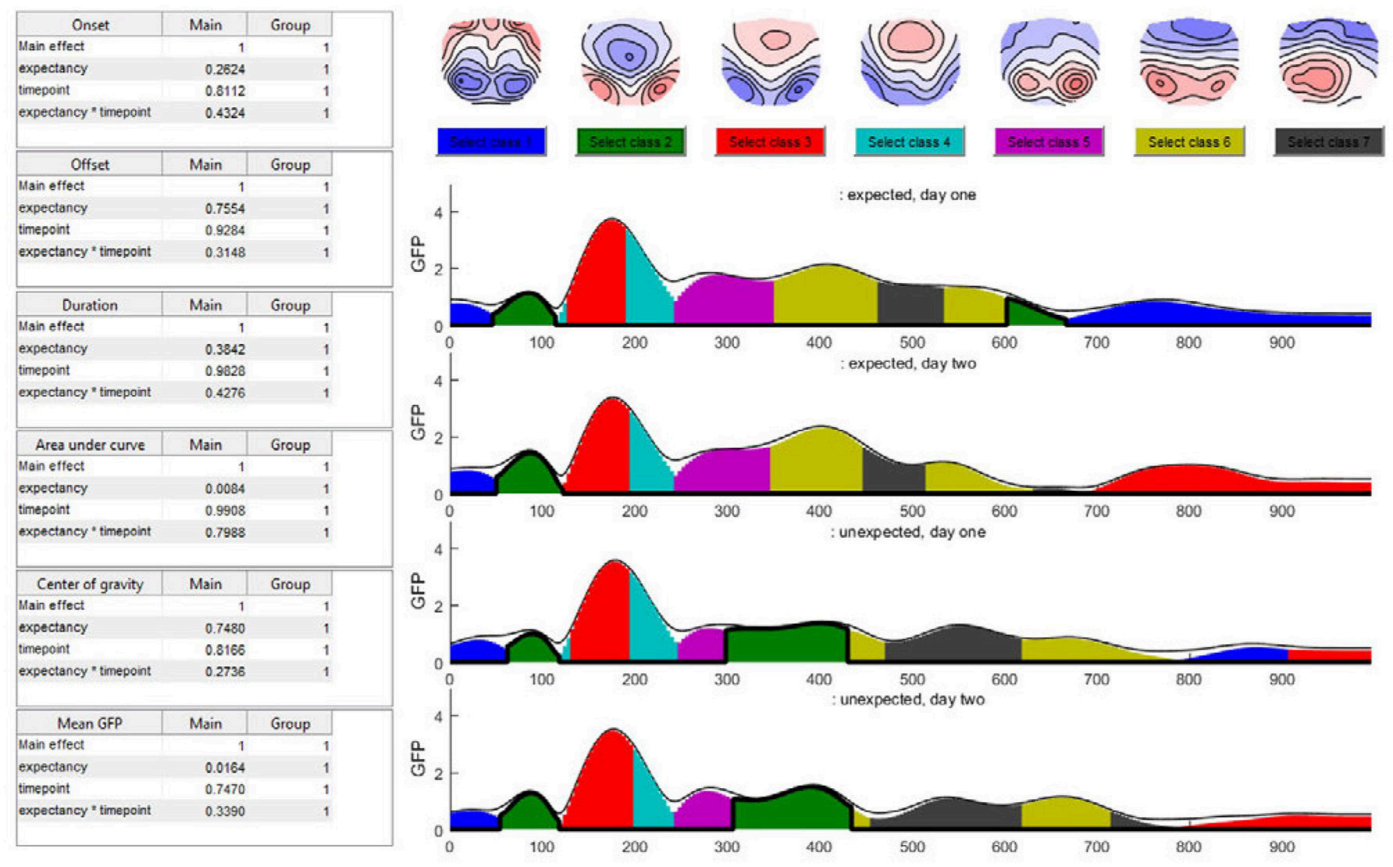
The seven-class solution of the microstate assignment for the four conditions (“expected vs. unexpected x day 1 vs. day 2”). The y-axis indicates the GFP-value while the x-axis indicates time (ms). The different colors stand for specific microstate classes, and the height of the colored areas indicate the GFP explained by the corresponding microstate map. The thin black line indicates the total GFP of the data. Note how well the different microstates explain the overall variance of the data. At the top of the graph, the potential maps of the different microstate classes are shown. The table on the left side shows the results of the selected microstate class fitting statistics, separated for each factor and the interaction effect. The parameters analyzed are the onset latency, offset latency, duration, area under the curve, center of gravity, and mean GFP of each microstate class. Note that in the current analysis, the selected microstate class two appears twice in time, and thus most likely represents two functionally different states with similar maps. To disentangle this, one may limit the analysis window for the microstate statistics to include only a certain period of interest.

In the time window around 400 ms after stimulus onset, the predominant microstate class in the unexpected sentence endings differed from that in the expected sentence endings. After 200 ms, this microstate class two (dark green color in Figure 15) was not observed during the expected sentence endings. The results indicate that the mean GFP and area under the curve (AUC) of microstate two are significantly different between the expected and unexpected sentence endings (GFP: *p* = 0.016; AUC: *p* = 0.008).

Additionally, the map of microstate two has a central negativity, which corresponds with results reported by Brandeis et al. (1995) for the N400 microstate map of incorrect sentence endings. There is also a strong significant effect of expectancy for the microstate class five (pink color in Figure 15) which starts around 240 ms after the stimulus onset in all conditions but ends earlier during unexpected sentence endings (Duration: *p* = 0.006; Offset: *p* = 0.02).

##### 4.4.4.2. Interaction effect

The TANOVA revealed an interaction effect in the time window between 580 and 640 ms after stimulus onset. Figure 15 shows that in this time window, the microstate classes seven (dark gray) and six (light green) are present in all the conditions. During the unexpected sentence endings, microstate seven lasted longer and microstate six had a later onset than during the expected sentence endings.

To specify the interaction effect that we found in the TANOVA (see Figure 10) we examine the differences between the expected sentence ending on day 1 and 2 in the interaction effect time window. On day 2, microstate seven is a bit shorter than on day 1. This could reflect a learning process, because there is no such difference between the two measurements of the unexpected sentence endings: they look quite similar. We may interpret this result as a learning effect which accounts only for the expected sentence endings. On day 1, the German words were relatively unfamiliar to the subjects, so microstate seven was nearly as long as that in the unexpected sentence endings. Later, as they improved their language skills, microstate seven became shorter. However, in the present small dataset, the statistics show neither significant differences in microstate six or seven nor significant interaction between the factors on the microstate level, such that this observation remains purely descriptive.

## 5. Discussion

The aim of this article was to illustrate the analysis of ERP data using randomization statistics as implemented in the MATLAB-based program Ragu, using an example study. It focused on the presentation of the different options and functions integrated in Ragu to familiarize students and more experienced scientists who want to analyze ERP data with a multivariate approach and to help them understand the underlying concepts of the tests and statistics. The discussed example study investigated the effects of language acquisition on the ERP reaction on expected and unexpected sentence endings in a foreign language before and after a 5-month long acquisition period. The results of the different test options of Ragu can now be integrated.

First, we tested if the stimuli employed in the study produced consistent ERP activity in the subjects at both measurement times. To do so, a TCT was computed. The TCT revealed significant consistency for all the conditions from the beginning of the data until almost the end of the analysis period. Interestingly, there was a time-period (after about 600 ms) where the TCT failed to yield evidence for a consistent map across subjects selectively in the expected sentence endings of the second measurement. For the unexpected sentence endings, there seemed to be no such differences between day 1 and 2. Having established that there is a consistent ERP activity in all conditions, it was reasonable to perform further data analysis.

The TANOVA tested for significant topographic differences in all time points of the data by comparing the GFP of difference maps in the different factor levels. We identified a rather long time period where the expected and unexpected sentence endings differed significantly, and also a short moment in time where day 1 and 2 differed significantly. In addition, we found an interaction effect between 580 and 640 ms after stimulus onset. To control for the effects of multiple testing, overall statistics for the count, duration, and Fisher's combined probability values were applied. Their outcome strengthened the validity of the significant result comparing the expected and unexpected sentence endings, but the effect on the day and the interaction effect were not significant in the overall statistics. However, assuming the presence of a pre-existing background information provided an external justification to expect these effects of day and interaction. For demonstration purposes, we continued to analyze the results concerning the interaction effect observed using the TANOVA.

For further interpretation of the late interaction effect, *post-hoc* tests were applied. The expected sentence endings as well as the unexpected sentence endings were compared between day 1 and 2. The t-maps showed a much stronger result for the comparison of the expected sentence endings between day 1 and 2, whereas the resulting t-map of the unexpected sentence endings was rather flat, indicating small *t*-values and no substantial difference between the different measurement time points of the unexpected sentence endings. Furthermore, the TANOVA revealed a significant difference when correct sentence endings between day 1 and 2 were compared. However, no significant difference was observed for false sentence endings between day 1 and 2 (see Figure 13). This supports a conclusion that at a rather late stage of processing, the learning effects were driven by the processing of correct sentence endings, which coincides with the observation made based on the TCT analysis.

Finally, we used microstate analysis as an additional analysis method to quantify the latency effects of the experimental manipulations. The difference between the levels of the factor “expectancy,” also visible in the TANOVA, can be interpreted as an N400 effect. In the time window about 400 ms after stimulus onset, we expected to find a microstate showing posterior negativity (Brandeis et al., 1995), which would not occur in the expected sentence endings. Indeed, we found a microstate reflecting the N400 map with a central negativity, and only occurring in the unexpected sentence endings around 300–420 ms after stimulus onset. This is evidence that another mental process was active if the ending of a sentence violated the semantic expectancy of the subjects, as compared to the congruent case. However, no significant result for the interaction effect was found in any of the analyzed microstate classes. Therefore, based on the microstate analyses that we have conducted, we cannot argue in favor of a learning effect between day 1 and 2 regarding the expected sentence endings. However, the advantage is that by using the microstate approach we can gain information on the topographic nature and latency of short duration brain events. Taken together, it becomes apparent that all the results of the different tests in Ragu pointed to a similar story. We already saw differences in the TCT that were buttressed by the TANOVA and *post-hoc* tests.

## 6. Conclusion

We aimed to demonstrate useful features of Ragu. Ragu implements a series of analysis tools for evaluating experimental ERP data with meaningful and valid statistics, but without being dependent of a-priori models. The user does not have to specify a set of channels, time window, or type of inverse solution to begin with. However, Ragu provides an overall view of the time course and effects of the different groups, and factor levels in the entire data. Similar to other open source programs, Ragu provides a democratic approach for conducting scientific research. It can be used and adapted for specific purposes by a broad range of scientists. We hope that the present article provides an easy-to-grasp guide into the applications of the tool and we look forward to receiving positive feedback on its functionality.

## Author contributions

All authors listed have made a substantial, direct and intellectual contribution to the work, and approved it for publication.

### Conflict of interest statement

The authors declare that the research was conducted in the absence of any commercial or financial relationships that could be construed as a potential conflict of interest.

## Footnotes

^1^This paper was written by students and for students. The emphasis is thus on concrete and hands-on procedures and data-based explications, and not on the more abstract mathematical principles. For more general information about the underlying algorithms, please refer to the methods in the corresponding papers cited in this article.

^2^Compared to a previous paper demonstrating the usage of the software (Koenig et al., 2011), the present paper additionally covers the outlier detection, the topographic consistency test, *post-hoc* comparions and the microstate analysis.

^3^The study was approved by the Ethics Committee of the Canton of Bern, and all participants gave their written informed consent.

^4^For a set of time-points assigned to a particular microstate class, the AUC is simply defined as the sum of the GFP values of those time-points. The center of gravity is defined as

(3) $$\begin{array}{l} C=\frac{\overset{n}{\underset{j=1}{\sum }}\left(GFP_{j}*t_{j}\right)}{\overset{n}{\underset{j=1}{\sum }}GFP_{j}}, \end{array}$$

where *n* is the number of time points assigned to the given microstate class, *GFP*_*j*_ is the GFP of the *j*-th time point assigned to the given class, and *t*_*j*_ is the latency of the *j*-th time point assigned to the given class.
